# The role of miR-134-5p in 7-ketocholesterol-induced human aortic endothelial dysfunction

**DOI:** 10.17179/excli2024-7342

**Published:** 2024-08-29

**Authors:** Kind-Leng Tong, Ahmad Syadi Mahmood Zuhdi, Pooi-Fong Wong

**Affiliations:** 1 Department of Pharmacology, Faculty of Medicine, Universiti Malaya, 50603 Kuala Lumpur, Malaysia; 2 Department of Medicine, Faculty of Medicine, Universiti Malaya, 50603 Kuala Lumpur, Malaysia

**Keywords:** miR-134-5p, 7-ketocholesterol, endothelial dysfunction, human aortic endothelial cells

## Abstract

Atherosclerotic cardiovascular diseases are the leading causes of morbidity and mortality worldwide. In our previous study, a panel of miRNA including miR-134-5p was deregulated in young acute coronary syndrome (ACS) patients. However, the roles of these ACS-associated miRNAs in endothelial dysfunction, an early event preceding atherosclerosis, remain to be investigated. In the present study, human aortic endothelial cells (HAECs) were treated with 7-ketocholesterol (7-KC) to induce endothelial dysfunction. Following treatment with 20 μg/ml 7-KC, miR-134-5p was significantly up-regulated and endothelial nitric oxide synthase (eNOS) expression was suppressed. Endothelial barrier disruption was evidenced by the deregulation of adhesion molecules including the activation of focal adhesion kinase (FAK), down-regulation of VE-cadherin, up-regulation of adhesion molecules (E-selectin and ICAM-1), increased expression of inflammatory genes (*IL1B*, *IL6 *and *COX2*) and AKT activation. Knockdown of miR-134-5p in 7-KC-treated HAECs attenuated the suppression of eNOS, the activation of AKT, the down-regulation of VE-cadherin and the up-regulation of E-selectin. In addition, the interaction between miR-134-5p and *FOXM1* mRNA was confirmed by the enrichment of *FOXM1* transcripts in the pull-down miRNA-mRNA complex. Knockdown of miR-134-5p increased *FOXM1* expression whereas transfection with mimic miR-134-5p decreased FOXM1 protein expression. In summary, the involvement of an ACS-associated miRNA, miR-134-5p in endothelial dysfunction was demonstrated. Findings from this study could pave future investigations into utilizing miRNAs as a supplementary tool in ACS diagnosis or as targets for the development of therapeutics.

## Introduction

Adhesion and aggregation of monocytes on endothelial cells and the development of foam cell lesions lead to the formation of atherosclerotic lesions. Rupture of vulnerable plaque and superimposed thrombosis with or without distal embolization cause atherothrombotic coronary occlusion. Incomplete coronary occlusion may lead to acute coronary syndromes while complete coronary occlusion will cause acute myocardial infarction.

Oxidized low-density lipoprotein (ox-LDL) is one of the major factors in the development of atherosclerosis (Cominacini et al., 2000[10]). Increased level of ox-LDL is found in atherosclerotic lesions and it is well-established that dyslipidemia-induced dysfunction of vascular endothelial cells is a critical step in the early stage of atherosclerosis (Le Master and Levitan, 2019[22]). Oxysterols, the products of oxidized cholesterol are formed either by auto-oxidation, enzymatically or by both processes. Ox-LDL contains mainly lysophosphatidylcholine (LPC), lipid ester-bound aldehydes, 7-ketocholesterol (7-KC), 7α-hydroxycholesterol, 7β-hydroxycholesterol, 5α,6α-epoxycholesterol, 5β,6β-epoxycholesterol, 25-hydroxycholesterol, (25R)-26-hydroxycholesterol, and cholesta-3,5-dien-7-one (Bourdon et al., 2000[5]). 7-KC is one of the most studied pro-inflammatory oxysterols in atherosclerosis, whereby it has more atherosclerotic activity than cholesterol (Oh et al., 2016[32]). In particular, 7-KC has been reported to (1) disrupt the barrier function of endothelial cells (Chalubinski et al., 2020[7]), leading to LDL deposition in the sub-endothelial space, (2) promoting the adhesion and transmigration of monocytes and leukocytes (Amaral et al., 2013[3]), and (3) inducing proliferation, migration and cell death of vascular smooth muscle cells (He et al., 2013[17]; Liao et al., 2010[26]). 

MicroRNAs (miRNAs) are a well-recognized group of short (≈22 nucleotides), non-coding RNAs. The role of miRNAs in gene regulation was demonstrated to mediate posttranscriptional downregulation of expression, translational repression and deadenylation-dependent decay of messages through partially complementary miRNA target sites in mRNA untranslated regions (UTR) (Wu et al., 2006[41]). MiRNAs are expressed and regulate many biological functions including tissue development, cell proliferation, cell division, cell differentiation, metabolism, apoptosis and protein secretion (Kabekkodu et al., 2018[19]). Perturbation of miRNA expression levels is significantly correlated with pathological conditions, diseases of different origins and malignancies (Kaur et al., 2020[20]). Disease-associated miRNAs hold promise as potential targets for the development of novel miRNA-based diagnostic, prognostic or therapeutic biomarkers (Vavassori et al., 2022[37]). 

Next gene sequencing result from our previous study showed deregulated levels of circulating miR-183-5p, miR-15a-5p, let-7i-5p and miR-134-5p in patients with acute coronary syndromes compared to healthy controls (Tong et al., 2018[36]). Elevation of miR-134-5p was reported with increased calcium deposition in vascular smooth muscle cells (Choe et al., 2020[9]) and stimulation of cardiomyocyte cells apoptosis (Xiao et al., 2019[42]). MiR-134-5p has been shown to accelerate atherosclerosis by promoting lipid accumulation and inflammatory responses in macrophages by up-regulating lipoprotein lipase (Lan et al., 2016[21]), accounting for its higher (3.5-fold) expression in peripheral blood mononuclear cells isolated from unstable angina patients (Hoekstra et al., 2010[18]). However, its role in endothelial dysfunction remains unclear. The present study aimed to investigate the role of miR-134-5p in 7-KC-mediated endothelial dysfunction in aortic endothelial cells.

## Materials and Methodology

### Cell culture

Human aortic endothelial cells (HAECs) were purchased from ScienCell Research Laboratories (California, USA). HAECs were maintained at 37 °C in a humidified atmosphere of 5 % CO_2_ in the air and cultured in complete endothelial cell medium (ECM; ScienCell Research Laboratories, California, USA) supplemented with 5 % v/v fetal bovine serum (FBS), 1 % v/v endothelial cell growth supplement (ECGS) and 1 % v/v penicillin-streptomycin. Young HAECs in passages 6 to 9 were used in subsequent experiments. 

### Real-time cell proliferation analysis

Real-time cell growth kinetics of 7-KC-treated HAECs were monitored using xCELLigence Real-Time Cell Analyzer (Agilent Technologies, California, USA) as previously described (Wong et al., 2017[39]). The measurement of cell growth is based on increasing electrical impedance as the number of adhered cells on the electronic plate (E-plate) surfaces increases, whereas decreased net adhesion due to smaller cell morphology and reduced cell number lower the electrical impedance. The impedance values are expressed as Cell Index (CI). Briefly, 50 µL of cell-free culture medium was added to each well of E-plate 96 and background reading was recorded. A cell suspension of 50 µL at a cell density of 5.0 x 10^3^ cells/well was then added into each well of E-plate 96 (Agilent Technologies, California, USA). When the cells entered the logarithmic growth phase, the cells were serum-starved for 6 hours prior to the treatment with 5, 10 and 20 µg/mL of 7-KC (Sigma-Aldrich, St. Louis, USA) and monitored for another 24 hours. The last CI measurement before the addition of 7-KC is designated as the reference time point (**t_ref_**). The CI at reference time point (t) was recorded (CI_tref_). Calculation of normalized CI (NCI_t_) at any time point after the addition of 7-KC was performed by dividing the cell index at the time point after 7-KC addition (CI_t_) by the cell index at the reference time point (CI_tref_). 

NCI_t _= CI_t_ / CI_tref_

**NCI_t_** is the NCI at timepoint **t, CI_t _**is the cell index of 7-KC-treated cells at time point **t**, and **CI_tref _**is the cell index at the reference time point **t_ref _**(last measurement before 7-KC addition).

### Endothelial nitric oxide synthase (eNOS) activation in HAECs 

Briefly, HAECs were seeded into 6-well plates at cell density of 1.5 x 10^5^ cell/well and incubated overnight. HAECs were subjected to serum starvation for 6 hours prior to the treatment with 5, 10 and 20 µg/mL of 7-KC for another 24 hours. Calcium ionophore A23187 (Sigma-Aldrich, St. Louis, USA) at 2.5 µM was added onto the 7-KC-treated or vehicle control HAECs for another 1 hour to stimulate the phosphorylation of eNOS. Following treatment, the cells were washed twice with pre-chilled 1X phosphate buffered saline (PBS) buffer and collected for total protein extraction. 

### Protein extraction and immunoblotting analysis

Total cellular protein was extracted using radioimmunoprecipitation assay (RIPA) Lysis Buffer System (Santa Cruz Biotechnology, Texas, USA) which was supplemented with 1 % v/v phenylmethyl-sulfonyl fluoride (PMSF) solution, 1 % v/v sodium orthovanadate solution and 1 % v/v protease inhibitor cocktail solution. The cell lysates were incubated on ice for 30-60 minutes prior to centrifugation at 15,000 x g for 30 minutes at 4 °C. Lastly, supernatant containing the protein lysates were collected and kept at -20 °C until further use. An equal amount of protein lysates was prepared in 5X sample buffer which contained 250 mM Tris-HCl (pH 6.8), 10 % SDS, 50 % v/v glycerol, 5 % β-mercaptoethanol, 0.1 % bromophenol. The protein lysates were separated by electrophoresis on 7.5 % to 10 % sodium dodecyl sulfate-polyacrylamide gel electrophoresis (SDS-PAGE) and then electro-transferred onto polyvinylidene fluoride (PVDF) membrane (Merck Millipore, Massachusetts, USA). The PVDF membrane was then blocked and probed with respective primary antibodies overnight at 4 °C (Table 1(Tab. 1)). Next, the PVDF membrane was washed and incubated with horseradish peroxidase-conjugated secondary antibodies. The protein-antibodies complexes were then visualized using the Enhanced Chemiluminescence Western Blotting Detection Kit (Cytiva Life Sciences, Amersham, UK). Densitometry analysis of the protein bands was performed using Quantity One software (Bio-Rad Laboratories, California, USA). Please refer to Supplementary data for all raw immunoblot images and protein intensities.

### Quantitative reverse transcription polymerase chain reaction (qRT-PCR) validation of miRNA and mRNA expression levels

For the mRNA and miRNA expression study, total RNA containing miRNA was isolated from HAECs using miRNeasy Mini Kit (QIAGEN Inc., Hilden, Germany) according to the manufacturer's instruction. Briefly, the cells in wells or flasks were washed twice with pre-chilled 1X PBS. The monolayer cells were lysed using QIAzol Lysis Reagent with scrapper. The cell lysates collected were further homogenized by vortexing for 1 minute and mixed vigorously with chloroform. The mixture was then centrifuged at 12,000 x g for 15 minutes at 4 °C. After centrifugation, the top aqueous layer containing RNAs was transferred to a new microcentrifuge tube before precipitating with 100 % ethanol. Next, the solution was passed through RNeasy Mini spin column and washed 3 rounds with buffer RWT and RPE. Finally, RNAs retained with small RNA species was eluted using RNase-free water and kept in -20 °C. The purity and yield of total RNA were determined using NanoDrop 2000 (Thermo Fisher Scientific, Massachusetts, USA). For quantification of miRNA expression, total RNA was reverse-transcribed using a TaqMan miRNA Reverse Transcription Kit (Applied Biosystems, Thermo Fisher Scientific, CA, USA) according to the manufacturer's instructions. Reverse transcription was performed under the following parameters: incubation at 16 °C for 30 minutes, followed by 42 °C for 30 minutes and 85 °C for 5 minutes. Validated TaqMan miRNA primers (Applied Biosystems, Thermo Fisher Scientific, CA, USA) for miR-183-5p (Assay ID: 002269), miR-134-5p (Assay ID: 001186), miR-15a-5p (Assay ID: 000389), let-7i-5p (Assay ID: 002221) were used for qRT-PCR. U6 snRNA (Assay ID: 001973) served as reference RNA for normalization of data. The mixture of cDNA and PCR reaction mix containing the validated Taqman miRNA primers were initially denatured at 95 °C for 20 seconds, followed by 40 cycles of denaturation at 95 °C for 1 second and both annealing and extension at 60 °C for 20 seconds respectively using StepOnePlus Real-Time PCR System (Applied Biosystems, Thermo Fisher Scientific, CA, USA).

For quantification of mRNA expression, cDNA was reverse-transcribed from total RNA using the High-Capacity RNA-to-cDNA kit (Applied Biosystems, Thermo Fisher Scientific, CA, USA). The mixture of RNA samples and reverse transcription reaction mix were incubated in a thermal cycler for 60 minutes at 37 °C, followed by 5 minutes at 95 °C. Subsequently, real-time PCR amplification was performed using PowerUp SYBR Green Master Mix (Thermo Fisher Scientific, Massachusetts, USA) and respective primers (Table 2(Tab. 2); Integrated DNA Technologies Inc., Iowa, USA) to determine the gene expressions of CAMP responsive element binding protein 1 (*CREB1*), forkhead box M1 (*FOXM1*)*,* lysine-specific demethylase 2A (*KDM2A*), cyclooxygenase-2 (*COX2*), interleukin-1β (*IL1B*) and interleukin-6 (*IL6*) in HAECs. Glyceraldehyde-3-phosphate dehydrogenase (*GAPDH*) (Qiagen QuantiTECT Primer Assay ID: Hs_GAPDH_1_SG) served as a housekeeping gene. The mixture of cDNA samples and PCR reaction mix were first held for 2 minutes at 50 °C and 2 minutes at 95 °C for UDG and DNA polymerase activation. The mixture was then subjected to 40 cycles of denaturation for 15 seconds at 95 °C and both annealing and extension for 1 minute at 60 °C. Cycle threshold (Ct) values were obtained after completion of amplifications and Ct values were normalized to *GAPDH*. Data analysis was performed using the 2^-ΔΔCt^ method (Livak and Schmittgen, 2001[28]). Log_2_-transformed normalized relative quantity which represents the expression of target miRNAs was used to plot graphs.

### miRNA knockdown in HAECs

MiRNA knockdown was performed by transfecting the cells with a short hairpin inhibitor. Transfection with miR-134-5p inhibitor (Dharmacon Inc., Horizon Discovery, Waterbeach, UK) was initiated 24 hours after HAECs were seeded onto 6-well plates (seeding density of 1.5 x 10^5^ cells per well) or T25 flasks (seeding density of 4.2 x 10^5^ cells per flask). MiRNA hairpin inhibitor Negative Control #2 (Dharmacon Inc., Horizon Discovery, Waterbeach, UK) was used as a non-targeting inhibitor control in miRNA inhibition experiments. After 6 hours of transfection, 5 % of FBS was added to the medium and the cells were incubated for an additional 18 hours. The cells were replenished with fresh complete ECM medium 24 hours post-transfection. Finally, the transfected cells were harvested for total RNAs and protein isolation at 24- and 48-hour post-transfection. Meanwhile, transfected cells were treated with 7-KC for another 24 hours. Following transfection, the cells were washed twice with pre-chilled 1X PBS and collected for total RNA and protein extraction.

### miRNA biotin-pull down assay

The association between miRNA and potential target mRNA was investigated using the biotinylated miRNA-mRNA complex pull-down assay. The formed miRNA-mRNA complex was isolated using magnetic streptavidin-conjugated beads through the binding of biotin and streptavidin. The efficiency of transfection of biotinylated miRNA mimic in HUVECs has been previously optimized in the laboratory (Wong et al., 2017[39]) and the HUVECs model enables the investigation of physiological and pathological impacts of various stimuli as it closely resembles human vascular endothelium (Medina-Leyte et al., 2020[29]). Therefore, this protocol was used in the present study. Briefly, transfection with biotinylated miR-134-5p (bi-miR-134-5p) mimic or biotinylated *C. elegans* miR-67 (bi-cel-miR-67) mimic (Dharmacon Inc., Horizon Discovery, Waterbeach, UK) was initiated 24 hours after HUVECs were seeded onto T25 flasks (seeding density of 4.2 x 10^5^ cells per flask). Cells transfected with bi-cel-miR-67 mimic served as non-targeting control. Dynabeads MyOne Streptavidin T1 (Invitrogen, Thermo Fisher Scientific, Massachusetts, USA) were washed thoroughly using washing buffer (10 nM Tris-HCl, pH7.5; 1 mM EDTA, pH 8; 2 M NaCl) and blocked in blocking buffer [NP-40 lysis buffer; 1 mg/mL BSA; 500 μg/mL yeast tRNA (Ambion, Invitrogen, Thermo Fisher Scientific, Massachusetts, USA)] at 4°C for 2 hours. The transfected cells were washed twice with pre-chilled 1X PBS and harvested by re-suspending with NP-40 lysis buffer [50 mM Tris-HCl, pH 8.8; 1 % NP-40; 150 mM NaCl; 50 U/mL RNaseOUT (Invitrogen, Thermo Fisher Scientific, Massachusetts, USA)]. The cytoplasmic lysate was isolated by centrifugation at 10,000 x g for 10 minutes at 4 °C. In the meantime, the blocked Dynabeads were washed using NP-40 lysis buffer and re-suspended in 100 μL of NP-40 lysis buffer. Dynabeads and cytoplasmic mixture were further incubated at 4 °C for 4-5 hours on a rotating mixer for target mRNA capture. The pull-down RNA (RNA bound to the beads) was isolated using RNeasy Mini Kit (QIAGEN Inc., Hilden, Germany) according to the manufacturer's RNA clean-up protocol. Enrichment ratio of pull-down lysate was calculated using the equation: Enrichment ratio = (Bi-miR-134-5p mimic PD/Bi-cel-miR-67 mimic PD)/(Bi-miR-134-5p mimic input/Bi-cel-miR-67 mimic input), where PD is the abbreviation for pull-down. 

### Statistical analysis

Data are shown as the means ± standard error of means (SEM). Statistical analysis was performed with Student's t-test or one-way analysis of variance (ANOVA) with Tukey's multiple comparison test using GraphPad Prism version 5.0 (GraphPad Software, California, USA), where applicable. The statistical significance of differences was accepted at *p*<0.05. 

## Results

### The effect of 7-KC treatment on endothelial cells

**Figure 1:**
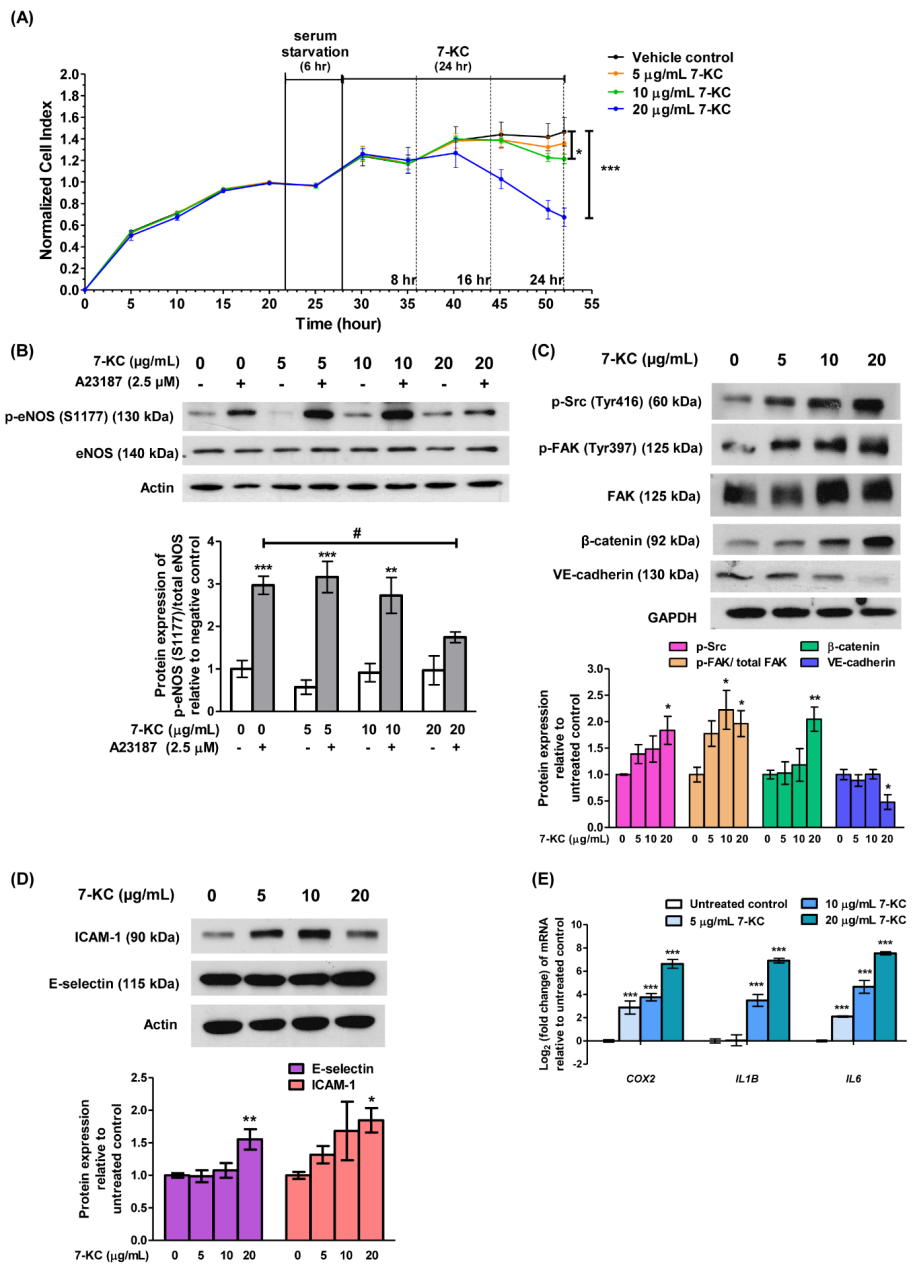
7-KC induces endothelial dysfunction in HAECs. (A) Sub-confluent HAECs were serum starved for 6 hours (hr) prior to the treatment with a range of concentrations (5, 10 and 20 μg/mL) of 7-KC for 8, 16 and 24 hr. The growth kinetics of 7-KC-treated HAECs was monitored in real-time using xCELLigence Real-Time Cell Analyzer. The impedance values were recorded and expressed as Cell Index. Data are expressed as means ± SD of triplicate wells. (B) Representative immune-blot images and densitometry analysis of phosphorylated eNOS [p-eNOS (S1177)] in 7-KC-treated HAECs with or without stimulation of A23187. A23187 is an activator of eNOS phosphorylation. Actin served as a loading control. Representative immune-blot images and densitometry analysis of (C) p-Src (Tyr416), p-FAK (Tyr397), β-catenin, VE-cadherin, (D) E-selectin and ICAM-1 in 7-KC-treated HAECs. GAPDH and actin served as loading control. (E) Log_2_ transformation of fold change of *COX2*, *IL1B, *and* IL6 *in HAECs treated with various concentrations of 7-KC for 24 hours. The expression of each mRNA was normalized to *GAPDH.* Data are shown as the means ± SEM of at least three independent experiments. Statistical analysis was performed using one-way ANOVA with Tukey's multiple comparison test and statistical significance is denoted as *, *p*<0.05, **, *p*<0.01, ***, *p*<0.001 compared to vehicle control without A23187; #, *p*<0.05 compared to HAECs treated with A23187 alone.

Endothelial dysfunction represents an early stage in the development of atherosclerosis and coronary artery disease. To establish endothelial dysfunction *in vitro*, endothelial cells were exposed to 7-KC, a primary oxysterol associated with atherosclerosis. Briefly, HAECs underwent 6-hour serum starvation, followed by incubation with increasing concentrations of 7-KC (5, 10 and 20 µg/mL). The growth kinetics of HAECs were closely monitored for an additional 24 hours. The real-time cell growth profile demonstrated a dose-dependent inhibition of HAECs growth by 7-KC (Figure 1A(Fig. 1); Supplement 1). Notably, treatment with 10 and 20 µg/mL of 7-KC resulted in significant growth inhibition, reducing cell growth by approximately 15 % and 50 %, respectively after 24 hours (Figure 1A(Fig. 1); Supplement 1). All three concentrations of 7-KC were subsequently employed in further experiments to investigate the role of miRNA in endothelial dysfunction.

NO plays an important role in regulating vascular function and pathophysiology and the production of NO is regulated by activation of eNOS. Endothelial dysfunction is characterized by diminished NO availability, which can result from a decrease in eNOS expression or impaired eNOS activation. To investigate the effect of 7-KC on the induction of endothelial dysfunction, A23187, a calcium ionophore was used to stimulate the phosphorylation of eNOS in HAECs. A23187, acting as an eNOS activator, significantly increased the expression of phosphorylated eNOS at Ser1177 in HAECs when compared to untreated control (Figure 1B(Fig. 1), lane 2 versus lane 1; Supplement 2). However, prior exposure of HAECs to 7-KC for 24 hours at 20 µg/mL but not 5 and 10 µg/mL, significantly inhibited the elevation of phosphorylated eNOS (Ser1177), in the presence of A23187 (Figure 1B(Fig. 1), lane 8 versus lane 2; Supplement 2). The inactivation of eNOS, hence, indicates endothelial dysfunction induced by 7-KC.

To further assess 7-KC-induced endothelial dysfunction, the expressions of endothelial adhesion and adherens junction proteins were examined. Phosphorylation of focal adhesion kinase (FAK) at Tyr397 and Src at Tyr416 leads to the dissociation of β-catenin from VE-cadherin and internalization of VE-cadherin, resulting in intercellular gap formation and an increase in endothelial permeability (Chen et al., 2012[8]). Treatment with 20 µg/mL 7-KC significantly increased the protein expression level of p-FAK (Tyr397), p-Src (Tyr416) and β-catenin but down-regulated the expression of adherens junction protein, VE-cadherin in HAECs (Figure 1C(Fig. 1); Supplement 3). On the other hand, the expressions of endothelial adhesion proteins, E-selectin and intercellular adhesion molecule 1 (ICAM-1) increased significantly by treatment of 20 µg/mL of 7-KC (Figure 1D(Fig. 1); Supplement 4). E-selectin and ICAM-1 were expressed by activated endothelial cells and play an important role in mediating endothelial-leukocyte adhesion (Gimbrone and García-Cardeña, 2016[15]). Furthermore, the exposure to 7-KC resulted in a significant elevation of mRNA expression of *COX2* and pro-inflammatory cytokines such as *IL1B* and *IL6 *(Figure 1E(Fig. 1)). Elevated expression of IL-1β and IL-6 is positively correlated with endothelial permeability (Alsaffar et al., 2018[1]; Puhlmann et al., 2005[33]), whereas the expression of COX-2 has been implicated in inflammation and is believed to contribute to the development of atherosclerosis (Burleigh et al., 2002[6]). Collectively, these results indicate the stimulation of endothelial damage upon treatment of 7-KC in HAECs. Hence, treatment of HAECs with 20 µg/mL of 7-KC was used to establish the endothelial dysfunction model in subsequent experiments.

### AKT activation in 7-KC-induced endothelial dysfunction in HAECs

**Figure 2:**
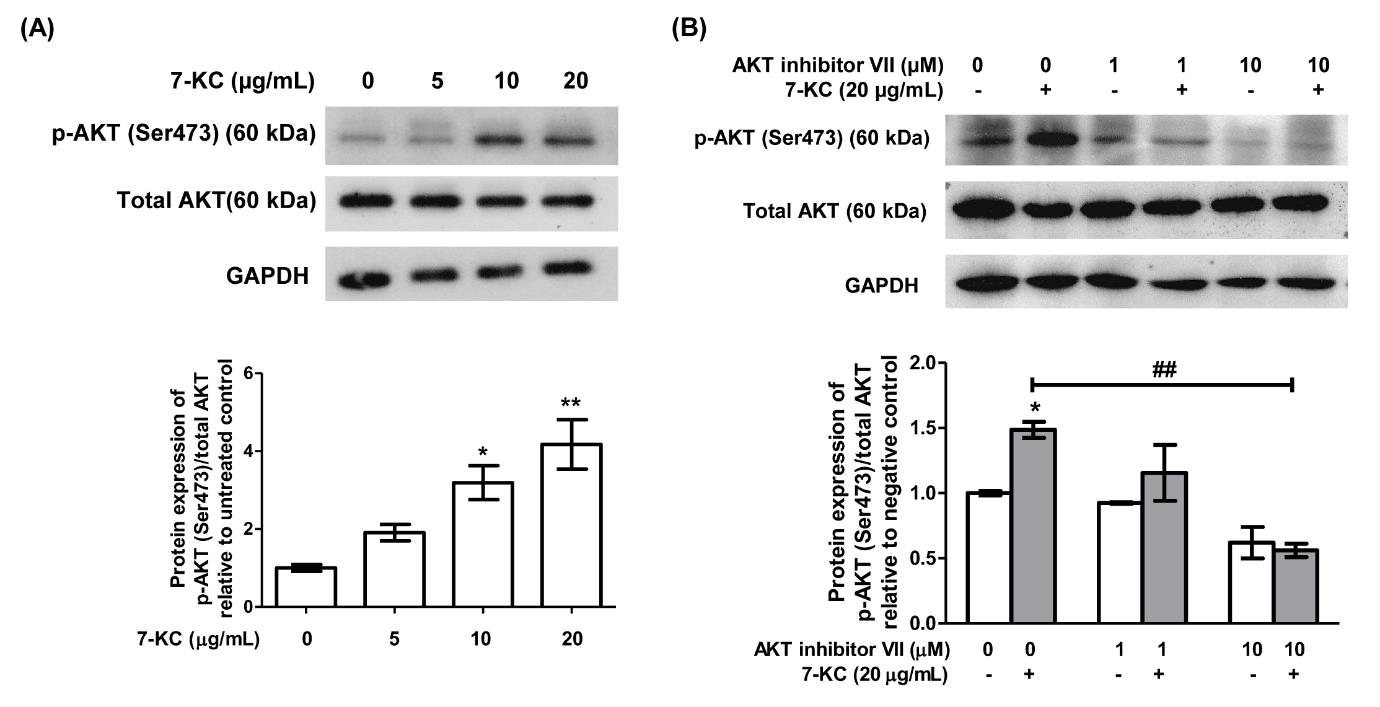
7-KC treatment activates AKT in HAEC. Representative immune-blot images and densitometry analysis of phosphorylated AKT [p-AKT (Ser473)] in HAECs treated with (A) 7-KC-treated alone or (B) in the presence of AKT inhibitor VII. GAPDH served as a loading control. Data are shown as the means ± SEM of three independent experiments. Statistical analysis was performed using one-way ANOVA with Tukey's multiple comparison test and statistical significance is denoted as *, *p*<0.05, **, *p*<0.01 compared to its respective untreated control; ##, *p*<0.01 compared to HAECs treated with 7-KC alone.

Numerous studies have documented the activation of AKT signaling by ox-LDL, including 7-KC in endothelial cells (Liao et al., 2010[26]; Zhu et al., 2021[49]). Treatment of 7-KC increased the protein level of p-AKT at Ser473 dose-dependently in HAEC after 24 hours (Figure 2A(Fig. 2); Supplement 5). Particularly, 10 and 20 µg/mL of 7-KC significantly up-regulated the expression of p-AKT (Ser473) in HAECs (Figure 2A(Fig. 2), lane 3 and 4; Supplement 5). The up-regulation of p-AKT (Ser473) by treatment of 20 µg/mL of 7-KC was attenuated in the presence of 1 and 10 µM AKT inhibitor VII (Figure 2B(Fig. 2), lane 4 and 6 versus lane 2; Supplement 6). This result confirmed the involvement of AKT activation in the induction of endothelial dysfunction by 7-KC in HAECs.

### Up-regulation of miR-134-5p by 7-KC treatment in endothelial cells

**Figure 3:**
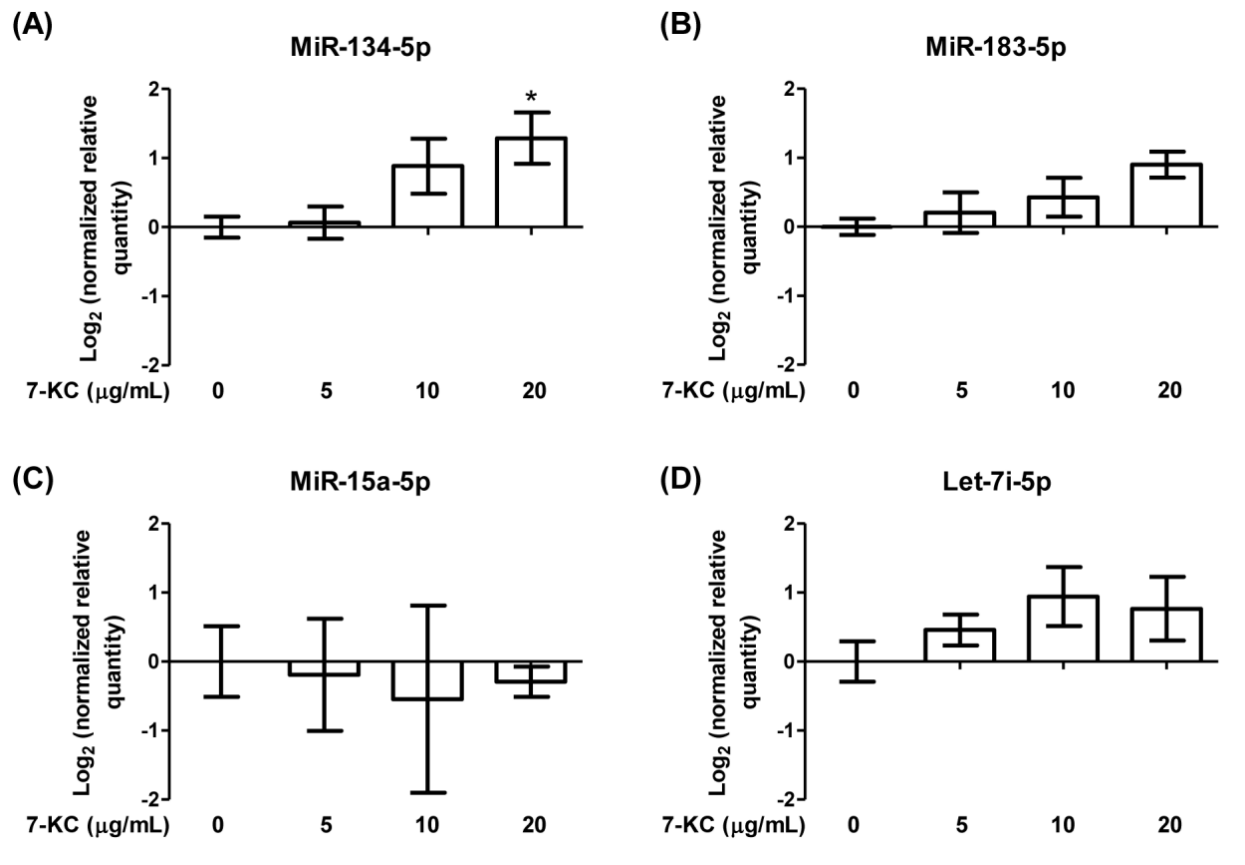
The expressions of target miRNAs in 7-KC-treated HAECs. Log_2_ transformation of normalized relative quantity of (A) miR-134-5p, (B) miR-183-5p, (C) miR-15a-5p and (D) let-7i-5p in 7-KC-treated HAECs. The expression of target miRNAs was normalized to U6 snRNA. Data are shown as the means ± SEM of three independent experiments. Statistical analysis was performed using one-way ANOVA with Tukey's multiple comparison test and statistical significance is denoted as *, *p*<0.05 compared to its respective untreated control.

A panel of deregulated miRNAs (miR-134-5p, miR-15a-5p, let-7i-5p and miR-183-5p) was identified in ACS patient plasma (Tong et al., 2018[36]), hence the expression levels of these miRNAs were further investigated in 7-KC-induced endothelial dysfunction model. MiR-134-5p was significantly up-regulated by 2.4-fold following 24-hour treatment with 20 µg/mL 7-KC in HAECs, whereas the expression levels of miR-183-5p, miR-15a-5p and let-7i-5p showed no significant changes in 7-KC-treated HAECs (Figure 3(Fig. 3)). Subsequently, the involvement of miR-134-5p in endothelial dysfunction was further investigated. 

### Knockdown of miR-134-5p attenuated 7-KC-induced endothelial dysfunction

**Figure 4:**
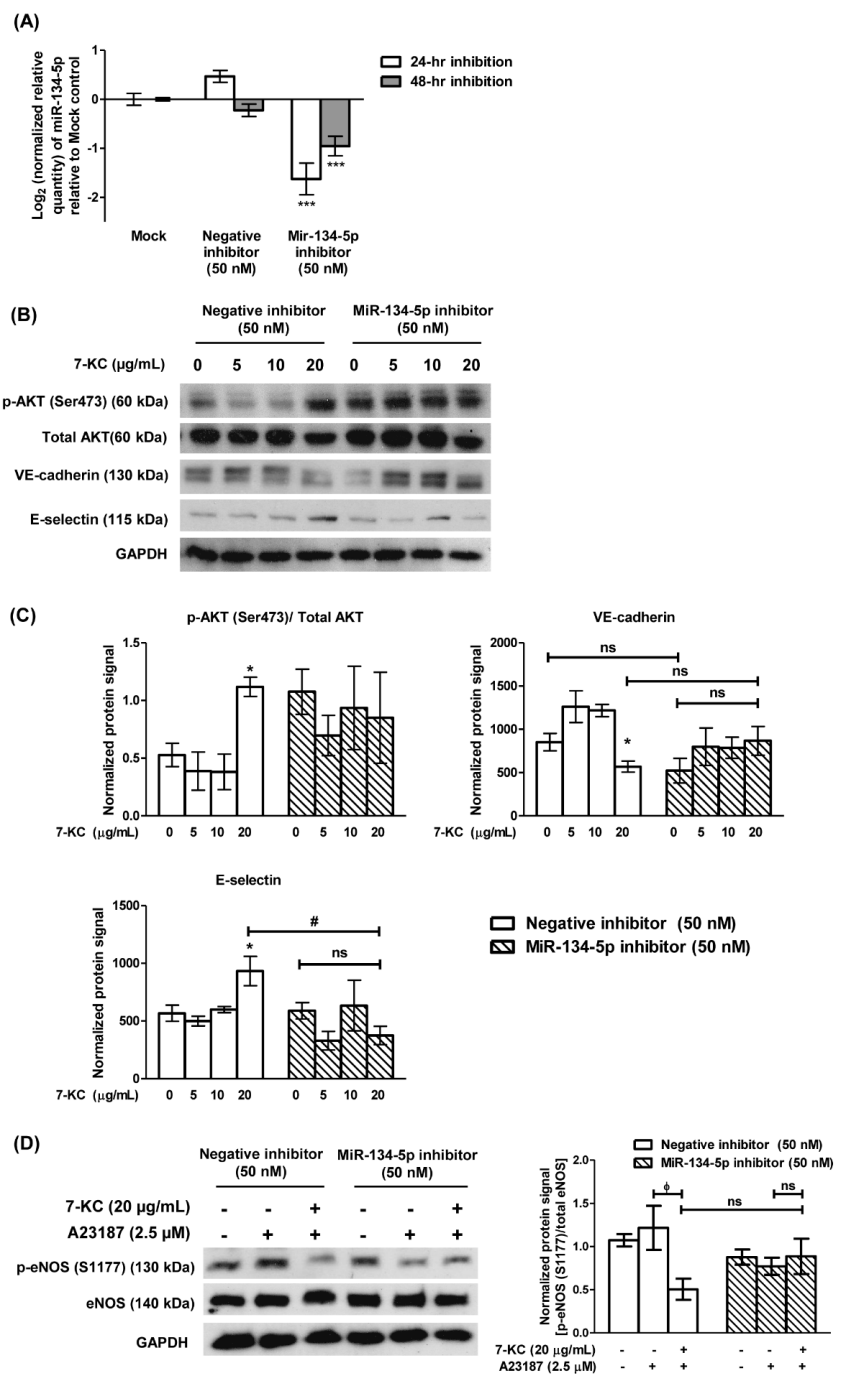
Transfection of miR-134-5p inhibitor attenuated 7-KC-induced endothelial dysfunction in HAECs. (A) Log_2_ transformation of the normalized relative quantity of miR-134-5p in HAECs 24- and 48-hour (hr) post-transfection. The mock sample was HAECs treated with 0.1 % transfection reagent without miR-134-5p inhibitor. The negative inhibitor served as a non-targeting control. The expression of miR-134-5p was normalized to U6 snRNA. HAECs were first transfected with 50 nM of negative inhibitor or miR-134-5p inhibitor for 24 hours and continued with 7-KC treatment for another 24 hours. (B) Representative immuno-blot images and (C) densitometry analysis of phosphorylated AKT [p-AKT (Ser473)], total AKT, VE-cadherin and E-selectin in 7-KC-treated miR-134-5p knockdown HAECs was demonstrated. (D) Representative immuno-blot images and densitometry analysis of p-eNOS (S1177) in 7-KC-treated miR-134-5p knockdown HAECs. A23187 was used to stimulate the phosphorylation of eNOS. The negative control inhibitor served as a non-targeting control. GAPDH served as a loading control. Data are shown as the means ± SEM from three independent experiments. Statistical analysis was performed using one-way ANOVA with Tukey's multiple comparison test and statistical significance is denoted as *, *p*<0.05, ***,* p*<0.001 compared to its respective mock sample or untreated control; #, *p*<0.05 compared to negative inhibitor transfection-HAECs treated with 20 μg/mL of 7-KC; ϕ, *p*<0.05 compared to respective A23187 alone sample. ns indicates not significant.

The involvement of miR-134-5p in 7-KC-induced endothelial dysfunction was investigated through a loss-of-function approach, by silencing miR-134-5p in HAECs using the miR-134-5p hairpin inhibitor or negative inhibitor control. Transfection of 50 nM miR-134-5p inhibitor into HAECs significantly down-regulated the level of miR-134-5p by 3-fold and 1.9-fold compared to the mock control in HAECs, at 24-hours and 48-hours post-transfection, respectively (Figure 4A(Fig. 4)). MiR-134-5p-knockdown HAECs were further treated with 7-KC for 24 hours. Treatment of 7-KC at 20 μg/mL retained the ability to increase p-AKT (Ser473) expression in HAECs transfected with negative inhibitor (Figure 4B(Fig. 4), lane 4 versus lane 1, and Figure 4C(Fig. 4), 4^th^ bar versus 1^st^ bar). Conversely, upon transfection with miR-134-5p inhibitor, there was no significant change in the expression level of p-AKT (Ser473) in cells treated with 20 μg/mL 7-KC, compared to untreated miR-134-5p knockdown HAECs (Figure 4B(Fig. 4), lane 8 versus lane 5, and Figure 4C(Fig. 4), 8^th^ bar versus 5^th^ bar). Additionally, in HAECs transfected with negative inhibitor, 7-KC significantly down-regulated VE-cadherin expression level compared to untreated non-targeting control HAECs (Figure 4B(Fig. 4), lane 4 versus lane 1, and Figure 4C(Fig. 4), 4^th^ bar versus 1^st^ bar). However, upon the knockdown of miR-134-5p in HAECs through miR-134-5p inhibitor, there was no significant change in the VE-cadherin level in cells treated with 20 μg/mL of 7-KC compared to its respective untreated cells (Figure 4B(Fig. 4), lane 8 versus lane 5, and Figure 4C(Fig. 4), 8^th^ bar versus 5^th^ bar) or 20 μg/mL of 7-KC-treated non-targeting control cells (Figure 4B(Fig. 4), lane 8 versus lane 4, and Figure 4C(Fig. 4), 8^th^ bar versus 4^th^ bar). Similarly, 20 μg/mL of 7-KC significantly increased the expression of endothelial leukocyte protein, E-selectin in HAECs transfected with negative inhibitor, compared to its respective untreated cells (Figure 4B(Fig. 4), lane 4 versus lane 1, and Figure 4C(Fig. 4), 4^th^ bar versus 1^st^ bar), however the up-regulated E-selectin expression was significantly decreased in the presence of miR-134-5p inhibitor in HAECs (Figure 4B(Fig. 4), lane 8 versus lane 4, and Figure 4C(Fig. 4), 8^th^ bar versus 4^th^ bar). Raw immunoblot images and protein intensities for Figure 4B and 4C(Fig. 4) are available in Supplementary 7. 

In the absence of miR-134-5p inhibitors, A23187 induced activation of eNOS (Figure 4D(Fig. 4), lane 2 versus lane 1; Supplement 8), however, pre-treatment of 7-KC significantly suppressed the expression of p-eNOS (S1177) induced by A23187 in HAECs (Figure 4D(Fig. 4), lane 3 versus lane 2; Supplement 8). Whereas in HAECs transfected with miR-134-5p inhibitor, the knockdown of miR-134-5p suppressed phosphorylation of eNOS induced by A23187 and resulted in an insignificant change in p-eNOS (S1177) level compared to its respective untreated cells (Figure 4D(Fig. 4), lane 5 versus lane 4; Supplement 8). Moreover, treatment with 7-KC at 20 μg/mL resulted in no significant change in the expression of p-eNOS (S1177) upon stimulation by A23187 in the miR-134-5p-knockdown HAECs (Figure 4D(Fig. 4), lane 6 versus lane 5; Supplement 8). Taken together, these results suggest that miR-134-5p modulates the expressions of the mediators of endothelial dysfunction in HAECs and its knockdown compromised this modulatory effect. 

### Identification of FOXM1 as the potential target gene of miR-134-5p

**Figure 5:**
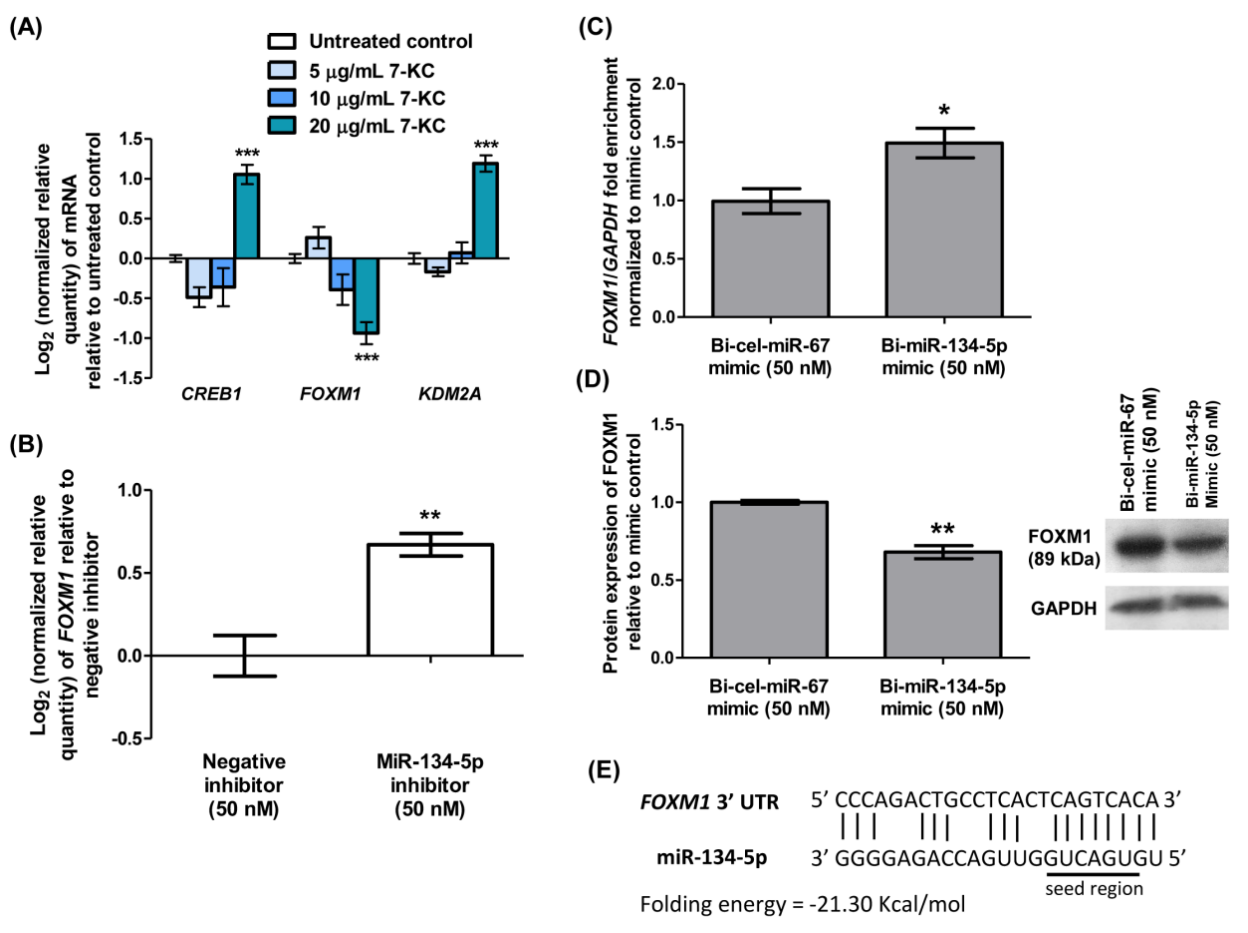
Identification of *FOXM1* as a potential target gene of miR-134-5p. (A) Log_2_ transformation of normalized relative quantity of *CREB1*, *FOXM1 *and* KDM2A* in HAECs treated with various concentrations of 7-KC for 24 hours. (B) Log_2_ transformation of normalized relative quantity of *FOXM1* in HAECs transfected with 50 nM miR-134-5p inhibitor for 24 hours. The negative inhibitor served as a non-targeting control. (C) Enrichment ratio of *FOXM1* normalized to *GAPDH* following magnetic bead pull-down of miR-134-5p-mRNA complex from HUVECs transfected with 50 nM biotinylated miR-134-5p (bi-miR-134-5p) mimic. Biotinylated *C. elegans* miR-67 (bi-cel-miR-67) mimic served as a non-targeting control. The expression of *FOXM1* was normalized to *GAPDH*. (D) Densitometry analysis and representative immuno-blot images of FOXM1 in HUVECs transfected with 50 nM bi-miR-134-5p or bi-cel-miR-67. (E) The predicted core binding sequence and folding energy of miR-134-5p in the 3'-UTR of *FOXM1 *by RNA22 v2. The solid line represents standard base pairing and strong interactions. The expression of each mRNA was normalized to *GAPDH.* GAPDH served as a loading control in immuno-blotting analysis. Data are shown as the means ± SEM from three independent experiments. Statistical analysis was performed using Student's t-test or one-way ANOVA with Tukey's multiple comparison test and statistical significance is denoted as *,* p*<0.05, **,* p*<0.01, ***,* p*<0.001 compared to respective untreated control or non-targeting control.

To identify the potential target gene of miR-134-5p, the mRNA expressions of several validated targets such as *CREB1 *(Yang et al., 2020[44]), *FOXM1 *(Wei et al., 2020[38]), and *KDM2A* (Li et al., 2020[24]), were determined by qRT-PCR from the total RNA of HAECs treated with 7-KC for 24 hours. Treatment of HAECs with 20 μg/mL 7-KC resulted in a significant up-regulation of *CREB1* and *KDM2A* mRNA expressions by 2-fold and the down-regulation of *FOXM1* by 2-fold (Figure 5A(Fig. 5)). In contrast, *FOXM1* was up-regulated by 1.6-fold in HAECs transfected with miR-134-5p inhibitor compared to those transfected with negative inhibitor (Figure 5B(Fig. 5)), indicating that miR-134-5p modulates *FOXM1*. Previous studies have validated *FOXM1* as a mRNA target of miR-134-5p using a luciferase reporter (Wei et al., 2020[38]). Here we used a biotinylated miRNA-mRNA complex pull-down assay to validate the association between miR-134-5p and *FOXM1*. Following the transfection of bi-miR-134-5p in HUVECs, an enrichment of *FOXM1* mRNA by 1.5-fold was obtained in the pull-down miRNA-mRNA complex compared to those transfected with bi-cel-miR-67 (Figure 5C(Fig. 5)). This result suggests the potential of *FOXM1* as a target gene of miR-134-5p. Translation repression of the target gene post-transcriptionally or degradation of target mRNA by miRNA leads to a down-regulation of the corresponding protein. Immunoblot in Figure 5D(Fig. 5) demonstrated significant down-regulation of FOXM1 protein expression by 1.5-fold in HUVECs transfected with bi-miR-134-5p mimic compared to those HUVECs transfected with bi-cel-miR-67 (Figure 5D(Fig. 5); Supplement 9). The predicted core binding sequence was analyzed using the online algorithm RNA22 v2 (Miranda et al., 2006[30]). A perfect complementary binding of miR-134-5p to the 3'-UTR of *FOXM1* at the seed region indicates the strong interaction between the miRNA and its target mRNA (Figure 5E(Fig. 5)). The heteroduplex between miR-134-5p and *FOXM1* scored a folding energy of -21.30 Kcal/mol (Figure 5E(Fig. 5)). The greater the negative folding energy value, the stronger the binding stability between the target mRNA and the miRNA binding sites (Alves et al., 2009[2]). Collectively, these results revealed the interaction between miR-134-5p and *FOXM1* in 7-KC-induced endothelial dysfunction in HAECs.

## Discussion

**Figure 6:**
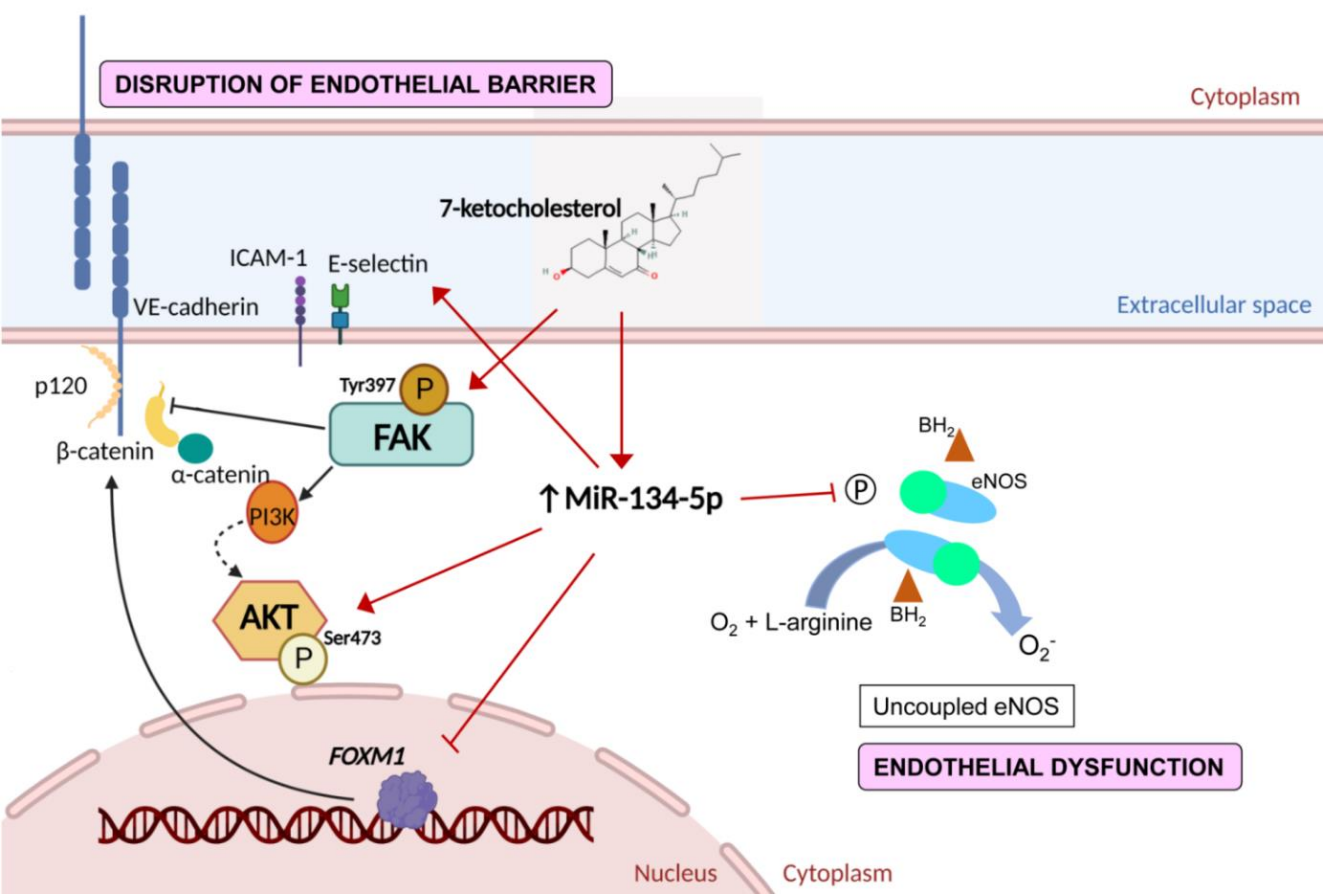
Schematic diagram of the effects of miR-134-5p in 7-ketocholesterol (7-KC)-induced endothelial dysfunction and disruption of endothelial barrier. Treatment of 7-KC suppressed eNOS activation, induced phosphorylation of focal adhesion kinase (FAK) at Tyr397, up-regulated β-catenin, down-regulated VE-cadherin, up-regulated adhesion molecules (ICAM-1 and E-selectin), as well as induced AKT activation. Increased expression of miR-134-5p was observed in 7-KC-treated endothelial cells. The interaction between miR-134-5p and FOXM1 mRNA was demonstrated in the present study. The diagram was drawn using BioRender.

Early alterations in endothelial function, including elevated plasma levels of soluble vascular adhesion molecules and increased vascular permeability, have been observed before the formation of atherosclerotic plaques in apolipoprotein E/low-density lipoprotein receptors (LDLR)-deficient mice (Bar et al., 2019[4]). Using 7-KC-induced endothelial dysfunction as a study model, the present study demonstrated that miR-134-5p is involved in endothelial dysfunction. Results showed that 7-KC triggered endothelial damage through the attenuation of eNOS activation by calcium ionophore A23187, activation of FAK, down-regulation of adherens junction protein VE-cadherin, as well as up-regulation of vascular adhesion proteins such as ICAM-1 and E-selectin in HAECs upon 24-hour treatment (Figure 6(Fig. 6)). VE-cadherin plays an important role in adherens junction formation, angiogenesis, and maintaining endothelial barrier and vascular integrity (Gavard and Gutkind, 2008[13]; Giannotta et al., 2013[14]). VE-cadherin recruits p120-catenin and β-catenin or plakoglobin (Dejana and Giampietro, 2012[11]) and through its cytoplasmic domain, bridges cadherin multimers to the actin cytoskeleton via actin-binding proteins such as α-catenin, vinculin and epithelial protein lost in neoplasm (Eplin). Activation of FAK and Src plays an important role in mediating vascular hyperpermeability (Guo et al., 2020[16]). Vascular endothelial growth factor A (VEGFA) promotes FAK activation, resulting in phosphorylation of β-catenin at Y142. This phosphorylation is associated with the dissociation of VE-cadherin/β-catenin and subsequent disruption of endothelial cell junction and increased endothelial permeability (Chen et al., 2012[8]). Increased expression of ICAM-1 and E-selectin in 7-KC-treated HAECs is in agreement with those reported in a study, whereby the up-regulation of adhesion molecules expression promotes the interaction of leukocytes and endothelial cells (Tani et al., 2018[35]). 

Several studies reported the activation of the AKT signaling pathway in endothelial cells induced by ox-LDL, particularly 7-KC and low shear stress (Zhang et al., 2017[46]; Zhu et al., 2021[49]). The protein kinase AKT is a major hub for several signal transduction pathways through its ability to phosphorylate numerous downstream targets directly involved in various cellular processes including proliferation, cell survival, and metabolism which is critical for vascular remodeling (Yu et al., 2015[45]). AKT1 is a well-established major kinase for eNOS-S1177 phosphorylation (Zhang et al., 2022[47]). A recent study revealed that AKT activation at Thr308, but not at Ser473 regulates p-eNOS S1177 and decreases NO production in HUVECs under physiological conditions (Liang et al., 2021[25]). This finding supports the observation of the present study where treatment of 7-KC significantly induced AKT activation (at Ser473) in HAECs without the activation of eNOS activity. In addition, the protective role of AKT phosphorylation at Serine 473 has also been reported to improve endothelial function experimentally in endothelial cells as well as hypertensive and diabetic murine models (Gao et al., 2017[12]; Wu et al., 2020[40]).

The present study also demonstrated the association of miR-134-5p and AKT-Ser473 phosphorylation in endothelial damage induced by 7-KC in HAECs (Figure 6(Fig. 6)). The involvement of miR-134-5p in mitogen-activated protein kinases (MAPK) and phosphoinositide 3-kinase (PI3K)/AKT signaling were reported in renal cell carcinoma, glioblastoma, and myocardial ischemia model (Xiao et al., 2019[42]; Zhang et al., 2014[48]). Inhibition of miR-134-5p attenuated (1) the 7-KC-induced phosphorylation of AKT (Ser473), (2) deregulation of VE-cadherin and E-selectin, and (3) ceased the inhibition effects of 7-KC on eNOS activation stimulated by calcium ionophore A23187 in HAECs. The interaction between *FOXM1* and miR-134-5p was demonstrated by the enrichment of *FOXM1* mRNA in miRNA-mRNA complex pulled down by biotinylated-miR-134-5p mimic. *FOXM1* is known as a pro-oncogene, protein encoded by this gene is a transcription activator involved in the regulation of cell proliferation and differentiation. Association between miR-134-5p and *FOXM1* was demonstrated previously in inhibiting epithelial-mesenchymal transition (EMT) in non-small cell lung cancer cells (NSCLC) (Li et al., 2017[23]) and modulating cancer cell differentiation and progression (Wei et al., 2020[38]). Similarly, the critical role of FOXM1 was described in endothelial cells in the restoration of endothelial barrier function and endothelial repair by transcriptionally activating β-catenin expression (Mirza et al., 2010[31]), as well as the promotion of endothelial-to-mesenchymal (EndMT) induced by transforming growth factor-β (TGF- β) through Smad2/3 and binds to Snail promoter (Song et al., 2019[34]). Endothelial cells undergo EndMT when stimulated by ox-LDL, oxidative stress, hypoxia, low shear stress and inflammation, which promotes disruption of VE-cadherin and disassembly of adherens junctions. This ultimately leads to the impairment of endothelial integrity and promotes plaque calcification, thinning of the fibrous cap and plaque instability (Libby et al., 2019[27]; Xu and Kovacic, 2023[43]). Furthermore, the decreased expression of VE-cadherin may be attributed to the action of phosphorylated FAK, leading to the dissociation of VE-cadherin/β-catenin complexes. Moreover, the up-regulation of β-catenin could be the result of the rescue mechanism or a negative feedback mechanism of FOXM1 (Mirza et al., 2010[31]) in facilitating the re-annealing of the endothelial adherens junction following exposure 7-KC. 

In summary, the present study has demonstrated the initiation of endothelial damage by 7-KC in concomitant with elevation of miR-134-5p level in endothelial cells. Knockdown of miR-134-5p diminished 7-KC-induced eNOS inactivation, AKT activation, down-regulation of VE-cadherin and up-regulation of E-selectin in endothelial cells. 

## Declaration

### Acknowledgments

This study was supported by the Ministry of Higher Education, Malaysia (Fundamental Research Grant Scheme No. FRGS/2/2013/ SKK01/UM/02/3); and University of Malaya/Ministry of Higher Education (UM/ MOHE) High Impact Research Grant (HIRG; account number, J-20001-73812). 

### Conflict of interest

The authors have no competing interests associated with the manuscript.

### Author contributions

K-LT, ASMZ and P-FW participated in experimental design, data interpretation, and manuscript preparation. K-LT performed experiments. All authors have read and approved the final manuscript. 

## Supplementary Material

Supplementary data

## Figures and Tables

**Table 1:**
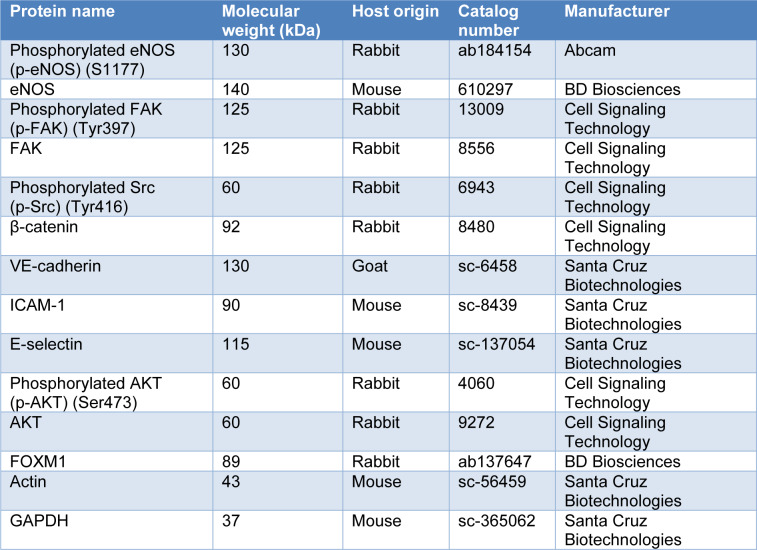
Details of antibodies used in immunoblotting analysis

**Table 2:**
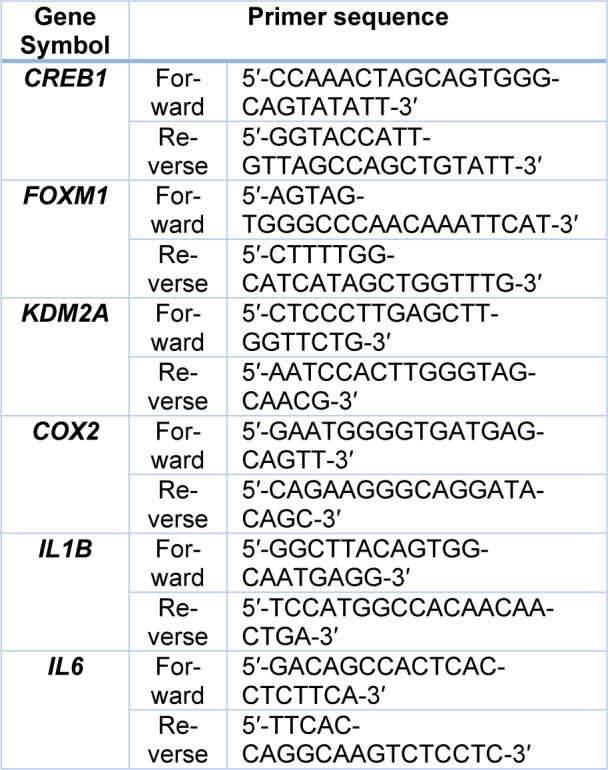
Primer pairs sequence utilized in mRNA qRT-PCR analysis
