# Supplementary material for: The role of miR-134-5p in 7-ketocholesterol-induced human aortic endothelial dysfunction
Source: EXCLI J. 2024 Aug 29;23:1073–90. doi: 10.17179/excli2024-7342 (PMC11464864; doi:10.17179/excli2024-7342)
Supplement: Supplementary data [file EXCLI-23-1073-s-001.pdf]

EXCLI Journal 2024;23:1073-1090 – ISSN 1611-2156
Received: May 03, 2024, accepted: August 22, 2024, published: August 29, 2024

Supplementary data to:

Original article:

THE ROLE OF MIR-134-5P IN 7-KETOCHOLESTEROL-INDUCED HUMAN AORTIC ENDOTHELIAL DYSFUNCTION

Kind-Leng Tong[1], Ahmad Syadi Mahmood Zuhdi[2], Pooi-Fong Wong[1]*

[1] Department of Pharmacology, Faculty of Medicine, Universiti Malaya, 50603 Kuala Lumpur, Malaysia
[2] Department of Medicine, Faculty of Medicine, Universiti Malaya, 50603 Kuala Lumpur, Malaysia

* **Corresponding author:** Pooi-Fong Wong, Department of Pharmacology, Faculty of Medicine, Universiti Malaya, 50603 Kuala Lumpur, Malaysia. E-mail: wongpf@um.edu.my

https://dx.doi.org/10.17179/excli2024-7342

This is an Open Access article distributed under the terms of the Creative Commons Attribution License (http://creativecommons.org/licenses/by/4.0/).

EXCLI Journal 2024;23:1073-1090 – ISSN 1611-2156
Received: May 03, 2024, accepted: August 22, 2024, published: August 29, 2024

Supplement 1: Raw data of Figure 1A

The cell growth profile of HAECs were monitored using xCELLigence Real-Time Cell Analyzer (RTCA; Agilent Technologies, California, USA). Briefly, HAECs were plated onto RTCA chamber and monitored overnight. Sub-confluent HAECs were then serum starved for 6 hours (hr) prior to the treatment with 7-ketocholesterol (7-KC). The treated cells were then monitored for another 24 hr. The growth curves were normalized to the Cell Index of the last measured time point before the addition of 7-KC or vehicle control.

Experiment 1

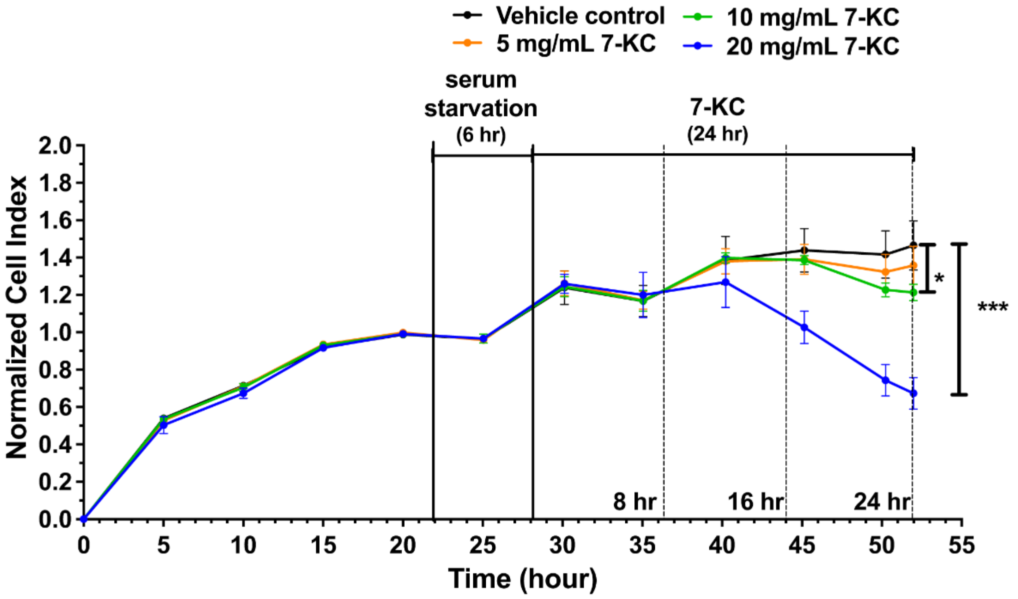

Table 1: Cell growth profile of HAECs treated with 7-ketocholesterol (7-KC) was monitored and Normalized Cell Index of HAECs were recorded. Normalized Cell Index is expressed as mean ± SD from triplicate wells.

Time (hour)	7-KC (µg/mL)			
	-	5	10	20
0	0.000	0.000	0.000	0.000
5	0.538 ± 0.008	0.528 ± 0.008	0.537 ± 0.018	0.503 ± 0.044
10	0.709 ± 0.007	0.710± 0.009	0.705 ± 0.021	0.674 ± 0.028
15	0.928 ± 0.004	0.936± 0.009	0.931 ± 0.014	0.917 ± 0.020
20	0.995 ± 0.008	0.998± 0.005	0.990 ± 0.008	0.967 ± 0.004
25	0.961 ± 0.009	0.959 ± 0.015	0.967 ± 0024	0.967 ± 0.013
30	1.227 ± 0.079	1.262 ± 0.066	1.244 ± 0.054	1.260 ± 0.051
35	1.13 ± 0.075	1.171 ± 0.047	1.168 ± 0.056	1.201 ± 0.121
40	1.378 ± 0.118	1.381 ± 0.068	1.398 ± 0.027	1.269 ± 0.135
45	1.447 ± 0.138	1.390 ± 0.081	1.386 ± 0.023	1.026 ± 0.088
50	1.413± 0.147	1.324 ± 0.094	1.227 ± 0.037	0.763 ± 0.085
52	1.467 ± 0.165	1.358 ± 0.103	1.214 ± 0.045	0.673 ± 0.085

EXCLI Journal 2024;23:1073-1090 – ISSN 1611-2156
Received: May 03, 2024, accepted: August 22, 2024, published: August 29, 2024

Experiment 2

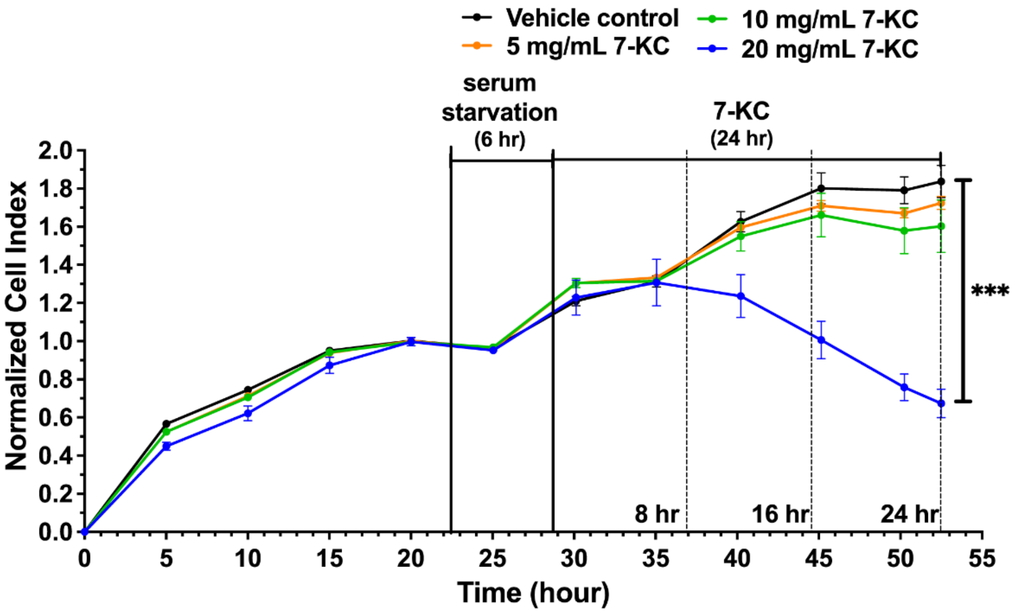

Table 2: Cell growth profile of HAECs treated with 7-ketocholesterol (7-KC) was monitored and Normalized Cell Index of HAECs were recorded. Normalized Cell Index is expressed as mean ± SD from triplicate wells.

Time (hour)	7-KC (µg/mL)			
	-	5	10	20
0	0.000	0.000	0.000	0.000
5	0.531 ± 0.013	0.524 ± 0.007	0.525 ± 0.002	0.449 ± 0.021
10	0.718 ± 0.021	0.712 ± 0.011	0.705 ± 0.007	0.622 ± 0.038
15	0.953 ± 0.007	0.939 ± 0.008	0.943 ± 0.003	0.873 ± 0.042
20	1.002 ± 0.008	1.000 ± 0.005	0.995 ± 0.008	0.998 ± 0.022
25	0.961 ± 0.014	0.961 ± 0.008	0.968 ± 0.016	0.953 ± 0.010
30	1.247 ± 0.007	1.303 ± 0.019	1.305 ± 0.024	1.228 ± 0.091
35	1.316 ± 0.027	1.334 ± 0.011	1.315 ± 0.017	1.307 ± 0.122
40	1.685 ± 0.031	1.596 ± 0.017	1.550 ± 0.077	1.236 ± 0.113
45	1.860 ± 0.043	1.710 ± 0.029	1.661 ± 0.114	1.006 ± 0.098
50	1.814 ± 0.059	1.671 ± 0.024	1.579 ± 0.120	0.758 ± 0.070
53	1.894 ± 0.056	1.724 ± 0.035	1.603 ± 0.137	0.674 ± 0.075

EXCLI Journal 2024;23:1073-1090 – ISSN 1611-2156
Received: May 03, 2024, accepted: August 22, 2024, published: August 29, 2024

Additional description of workflow in immunoblotting analysis. The protein lysates were separated on sodium dodecyl sulfate-polyacrylamide gel electrophoresis (SDS-PAGE) by electrophoresis. Protein lysates from two independent experiments were separated on single SDS-PAGE and then electro-transferred onto polyvinylidene fluoride (PVDF) membrane. The probed blot was imaged onto A4-sized X-ray film and then cropped to a smaller area for densitometry analysis.

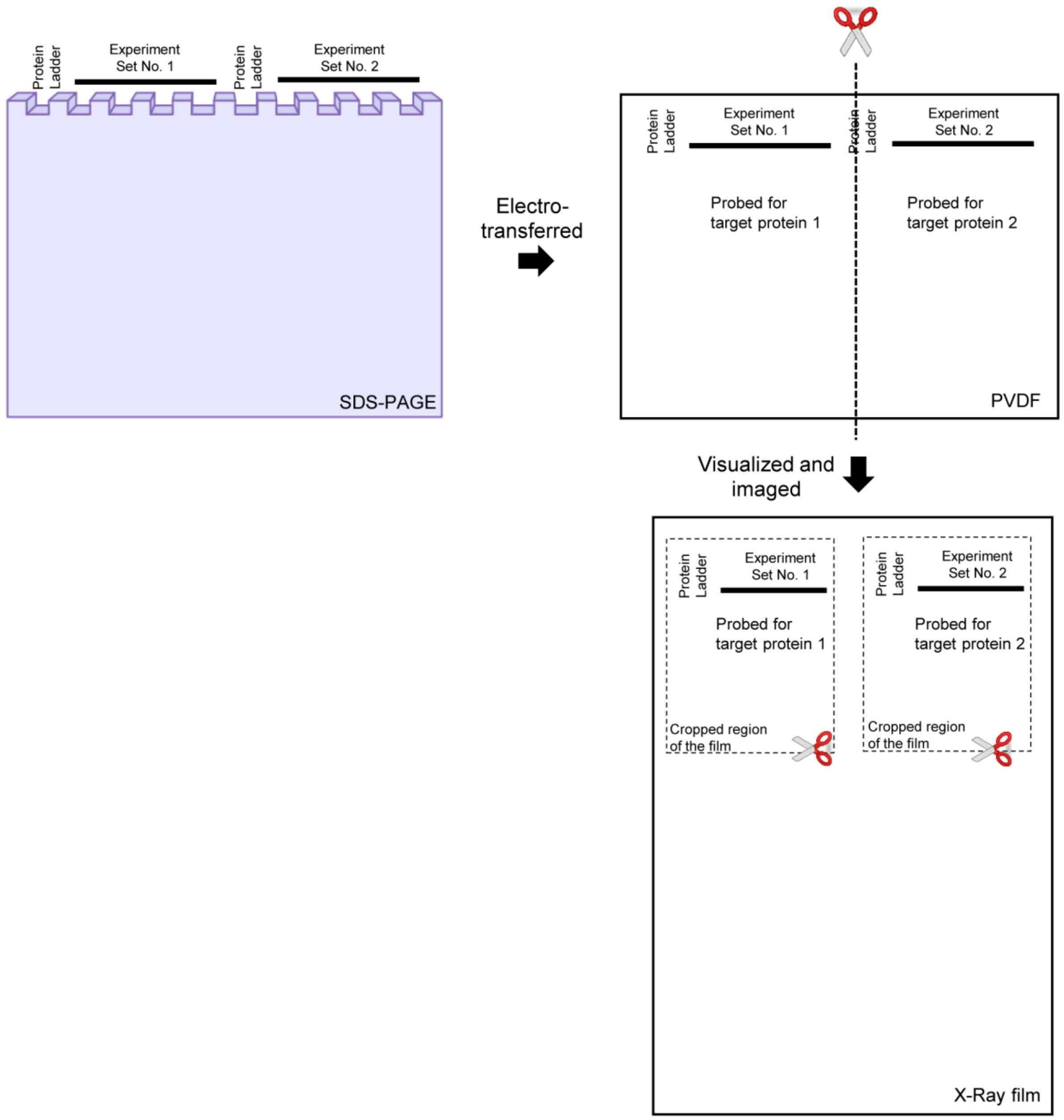

EXCLI Journal 2024;23:1073-1090 – ISSN 1611-2156
Received: May 03, 2024, accepted: August 22, 2024, published: August 29, 2024

Supplement 2: Raw immunoblot images and protein band intensities of Figure 1B

Immunobloting analysis of the expression of phosphorylated eNOS [p-eNOS (S1177)] and eNOS was performed for 7-ketocholesterol (7-KC)-treated HAECs with or without stimulation with A23187. A23187 is an activator of eNOS phosphorylation. Actin served as a loading control. Densitometry analysis of the expression of protein was quantified using Quantity One software (Bio-Rad Laboratories, California, USA) and shown in **Table 3**.

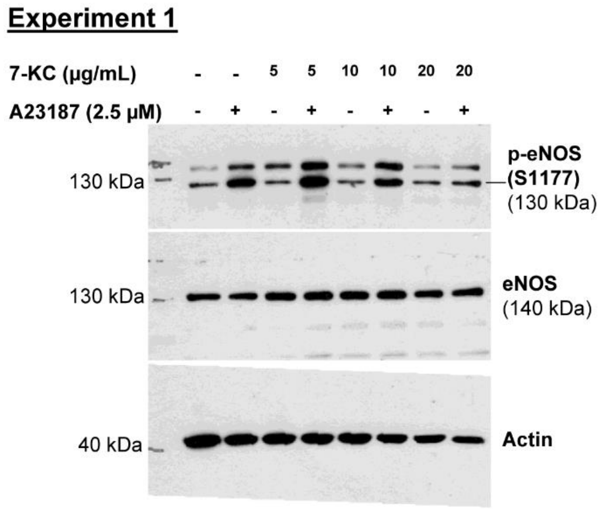

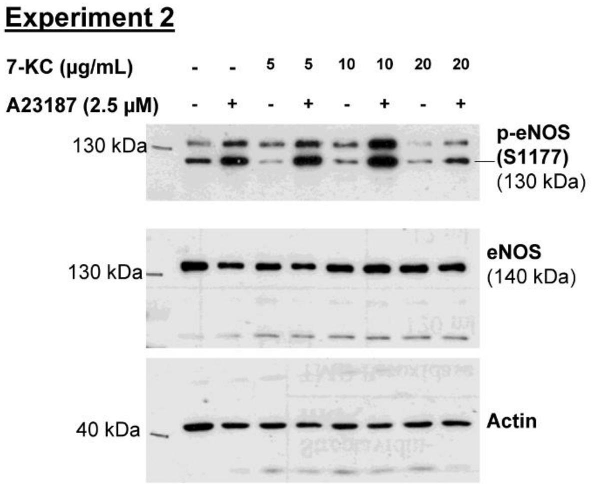

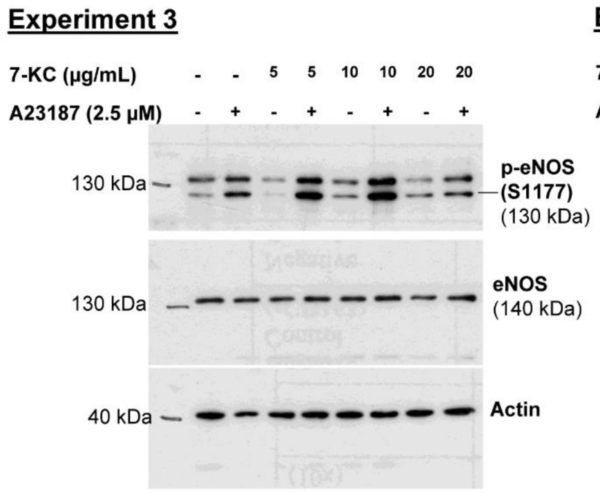

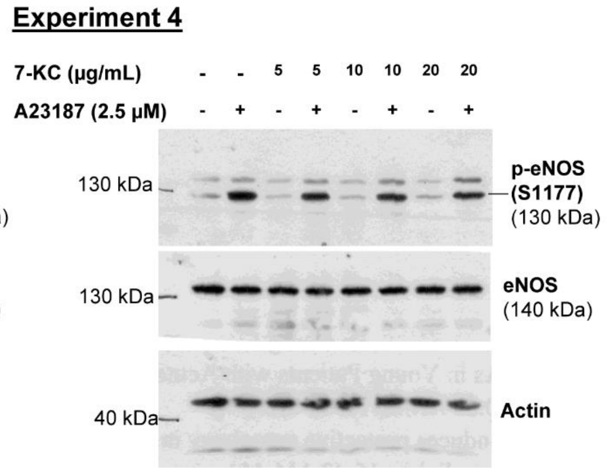

EXCLI Journal 2024;23:1073-1090 – ISSN 1611-2156
Received: May 03, 2024, accepted: August 22, 2024, published: August 29, 2024

Table 3: Expressions of p-eNOS (S1177) relative to total eNOS in 7-ketocholesterol (7-KC)-treated HAECs with or without stimulation with A23187.

		Protein band intensity normalized to its respective actin intensity in the same membrane*							
	**7-KC (µg/mL)**	-		5		10		20	
	**A23187 (2.5 µM)**	-	+	-	+	-	+	-	+
Experiment 1	p-eNOS (S1177)*	547.70	1400.70	575.10	1686.70	594.50	1641.90	739.10	1237.80
	eNOS*	1027.00	991.90	1254.30	1286.70	1312.30	1697.80	1576.10	1864.00
	p-eNOS / eNOS **(a)**	0.53	1.41	0.46	1.31	0.45	0.97	0.47	0.66
	**a/b**	1.24	3.29	1.07	3.05	1.05	2.25	1.09	1.55
Experiment 2	p-eNOS (S1177)*	721.20	1744.40	238.20	2025.30	525.80	1777.60	338.90	970.70
	eNOS*	1257.60	1149.90	1323.20	1347.40	1552.20	2038.00	1537.20	1478.40
	p-eNOS / eNOS **(a)**	0.57	1.52	0.18	1.50	0.34	0.87	0.22	0.66
	**a/b**	1.33	3.53	0.42	3.50	0.79	2.03	0.51	1.53
Experiment 3	p-eNOS (S1177)*	294.80	1182.60	123.00	1125.60	388.60	1295.80	433.90	641.20
	eNOS*	725.00	632.10	531.40	680.20	606.00	704.10	500.00	732.70
	p-eNOS / eNOS **(a)**	0.41	1.87	0.23	1.65	0.64	1.84	0.87	0.88
	**a/b**	0.95	4.35	0.54	3.85	1.49	4.28	2.02	2.04
Experiment 4	p-eNOS (S1177)*	234.30	1080.20	131.90	887.80	166.10	863.50	172.80	833.80
	eNOS*	1142.50	1222.10	1133.70	1026.10	1119.10	1073.40	1168.90	1380.70
	p-eNOS / eNOS **(a)**	0.21	0.88	0.12	0.87	0.15	0.80	0.15	0.60
	**a/b**	0.48	2.06	0.27	2.01	0.35	1.87	0.34	1.41
	Average p-eNOS/eNOS of untreated control **(b)**	0.43							
Plot graph	Average of **(a/b)** from 4 experiments	1.00	3.31	0.57	3.10	0.92	2.61	0.99	1.63

The background-adjusted intensity values of p-eNOS (S1177) and total eNOS were first normalized to their respective actin values of the same membrane. Next, the ratio of normalized p-eNOS (S1177) to total eNOS was calculated. This p-eNOS (S1177)/total eNOS ratio was then expressed relative to the average ratio obtained from untreated controls across four independent experiments and plotted accordingly.

EXCLI Journal 2024;23:1073-1090 – ISSN 1611-2156
Received: May 03, 2024, accepted: August 22, 2024, published: August 29, 2024

Supplement 3: Raw immunoblot images and protein band intensities of Figure 1C
Immunobloting analysis of the expression of (i) phosphorylated Src [p-Src (Tyr416)], (ii) p-FAK (Tyr397), (iii) β-catenin and (iv) VE-cadherin was performed for 7-ketocholesterol (7-KC)-treated HAECs. GAPDH served as loading control. Densitometry analysis of the expression of protein was quantified using Quantity One software (Bio-Rad Laboratories, California, USA).

(i) Phosphorylated Src [p-Src (Tyr416)]. Protein band intensity in **Table 4**

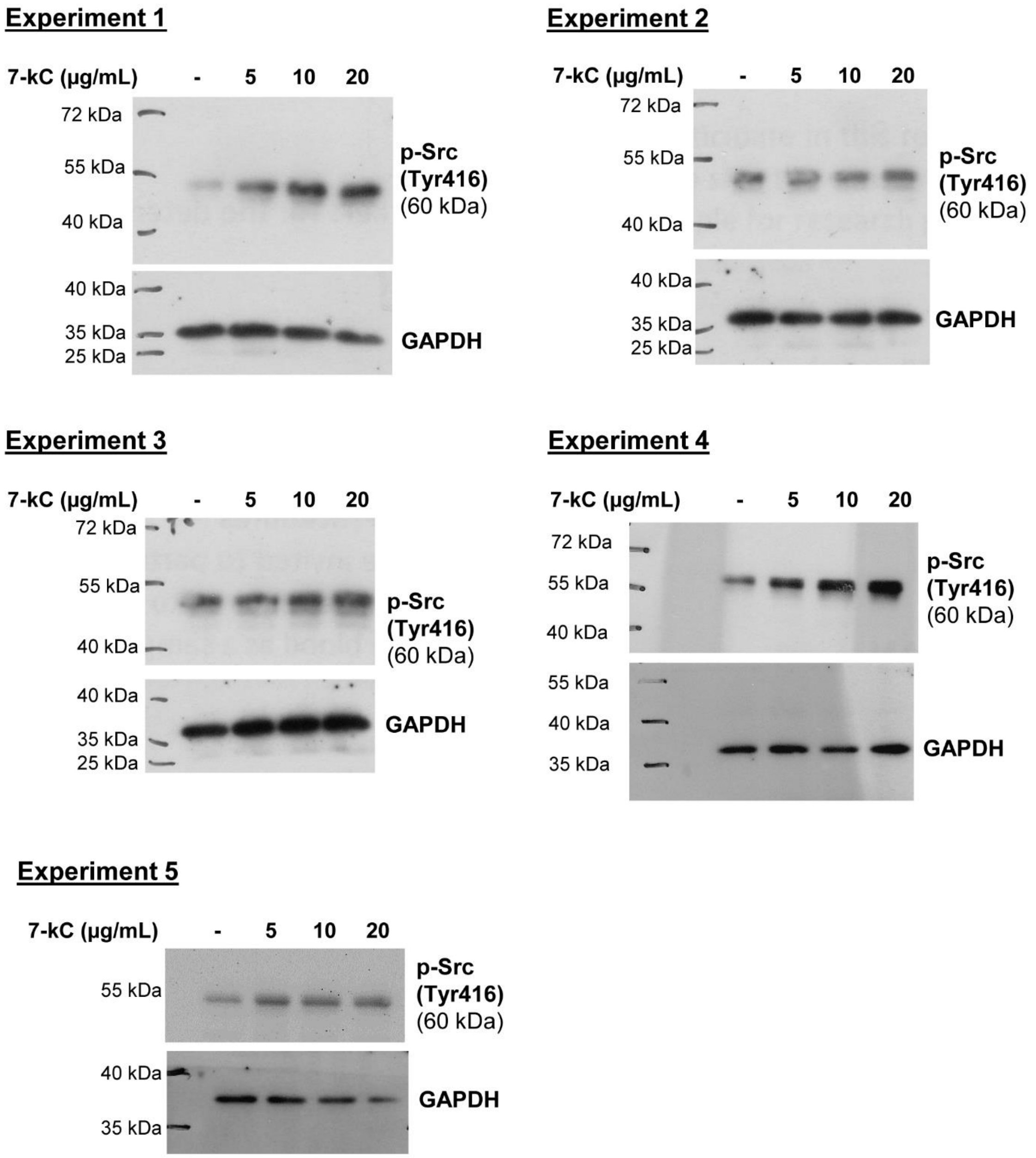

Table 4: Relative expression of **p-Src (Tyr416)** in 7-ketocholesterol (7-KC)-treated HAECs.

		Protein band intensity normalized to its respective GAPDH intensity in the same membrane **(a)**			
	**7-KC (µg/mL)**	-	5	10	20
Experiment 1	p-Src (Tyr416) **(a)**	987.60	1636.20	1451.10	2078.00
	**a/b**	1.02	1.68	1.49	2.14
Experiment 2	p-Src (Tyr416) **(a)**	978.30	772.60	851.60	1080.30
	**a/b**	1.01	0.79	0.88	1.11
Experiment 3	p-Src (Tyr416) **(a)**	1003.10	1139.30	1064.30	1379.10
	**a/b**	1.03	1.17	1.09	1.42
Experiment 4	p-Src (Tyr416) **(a)**	948.90	1706.30	1615.50	1855.00
	**a/b**	0.98	1.75	1.66	1.90
Experiment 5	p-Src (Tyr416) **(a)**	946.70	1501.90	2237.80	2542.60
	**a/b**	0.97	1.54	2.30	2.61
	Average p-Src (Tyr416) of untreated control **(b)**	972.92			
Plot graph	Average of **(a/b)** from 5 experiments	1.00	1.39	1.48	1.84

The background-adjusted intensity values of p-Src (Tyr416) were first normalized to their respective GAPDH values of the same membrane. The normalized p-Src (Tyr416) was then expressed relative to the average normalized values obtained from untreated controls across five independent experiments and plotted accordingly.

EXCLI Journal 2024;23:1073-1090 – ISSN 1611-2156
Received: May 03, 2024, accepted: August 22, 2024, published: August 29, 2024

(i) Phosphorylated FAK (p-FAK). Protein band intensity in **Table 5.**

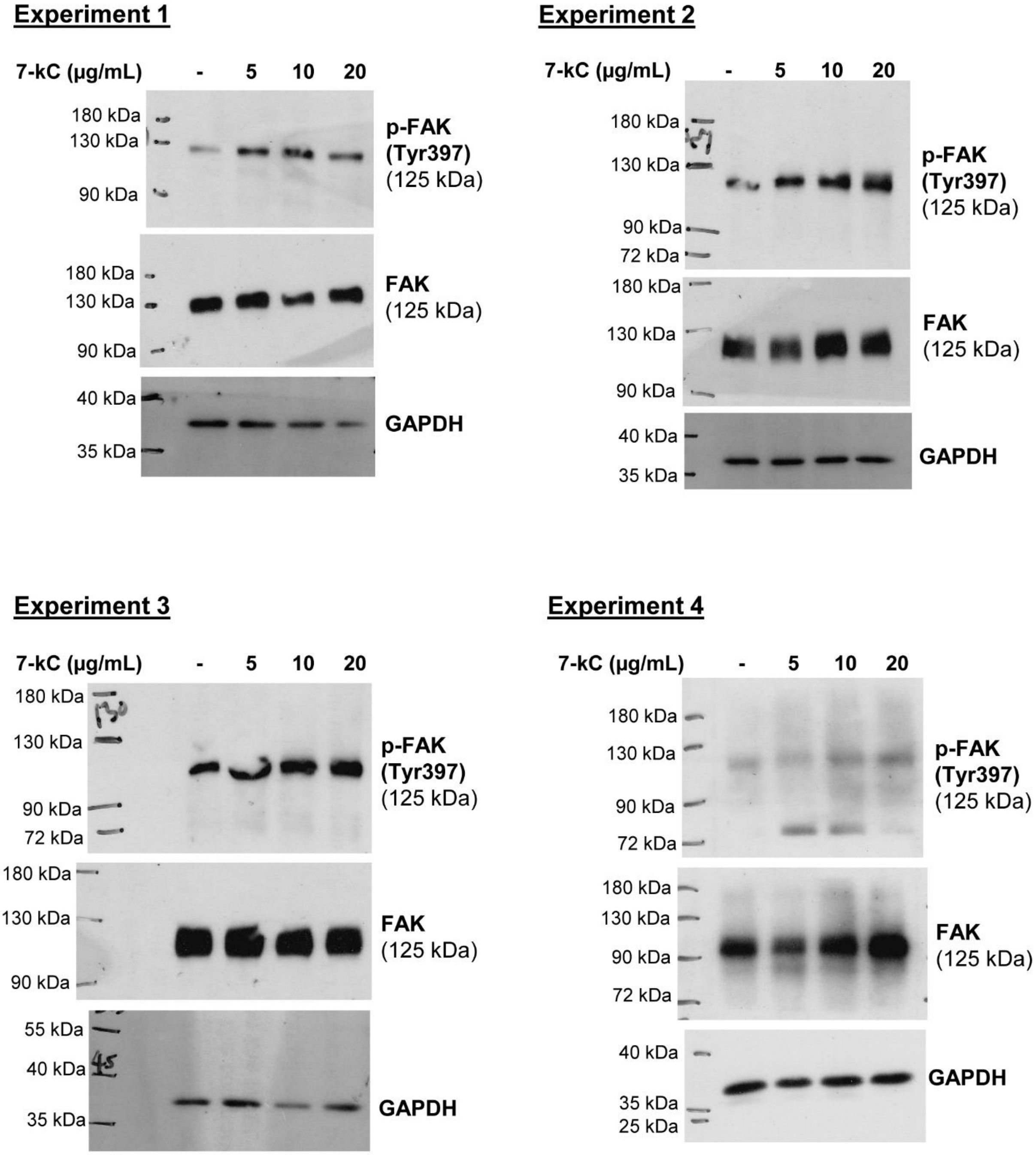

EXCLI Journal 2024;23:1073-1090 – ISSN 1611-2156
Received: May 03, 2024, accepted: August 22, 2024, published: August 29, 2024

Table 5: Expression of p-FAK (Tyr397) relative to total FAK in 7-ketocholesterol (7-KC)-treated HAECs.

		Protein band intensity normalized to its respective GAPDH intensity in the same membrane*			
	**7-KC (µg/mL)**	-	5	10	20
Experiment 1	p-FAK (Tyr397)*	208.50	572.80	652.00	487.90
	FAK*	1254.20	1342.40	906.90	1280.60
	p-FAK/FAK **(a)**	0.17	0.43	0.72	0.38
	**(a/b)**	0.66	1.69	2.84	1.51
Experiment 2	p-FAK (Tyr397)*	534.60	1138.90	1827.00	1506.10
	FAK*	1800.40	1630.70	2108.80	2101.60
	p-FAK/FAK **(a)**	0.30	0.70	0.87	0.72
	**(a/b)**	1.17	2.76	3.42	2.83
Experiment 3	p-FAK (Tyr397)*	869.40	923.20	1440.90	1250.20
	FAK*	2604.60	2189.20	3004.30	2229.50
	p-FAK/FAK **(a)**	0.33	0.42	0.48	0.56
	**(a/b)**	1.32	1.67	1.90	2.22
Experiment 4	p-FAK (Tyr397)*	609.60	820.10	968.10	1052.00
	FAK*	2836.10	2797.00	3416.00	2922.50
	p-FAK/FAK **(a)**	0.21	0.29	0.28	0.36
	**(a/b)**	0.85	1.16	1.12	1.42
	Average p-FAK/FAK of untreated control **(b)**	0.25			
Plot graph	Average of **(a/b)** from 4 experiments	1.00	1.82	2.32	1.99

Background-adjusted intensity values of p-FAK (Tyr397) and total FAK were first normalized to their GAPDH values from the same membrane. The ratio of normalized p-FAK (Tyr397) to total FAK was then calculated. This p-FAK (Tyr397)/total FAK ratio was then expressed relative to the average ratio obtained from untreated controls across four independent experiments and plotted accordingly.

EXCLI Journal 2024;23:1073-1090 – ISSN 1611-2156
Received: May 03, 2024, accepted: August 22, 2024, published: August 29, 2024

(ii) β-catenin. Protein band intensity in **Table 6.**

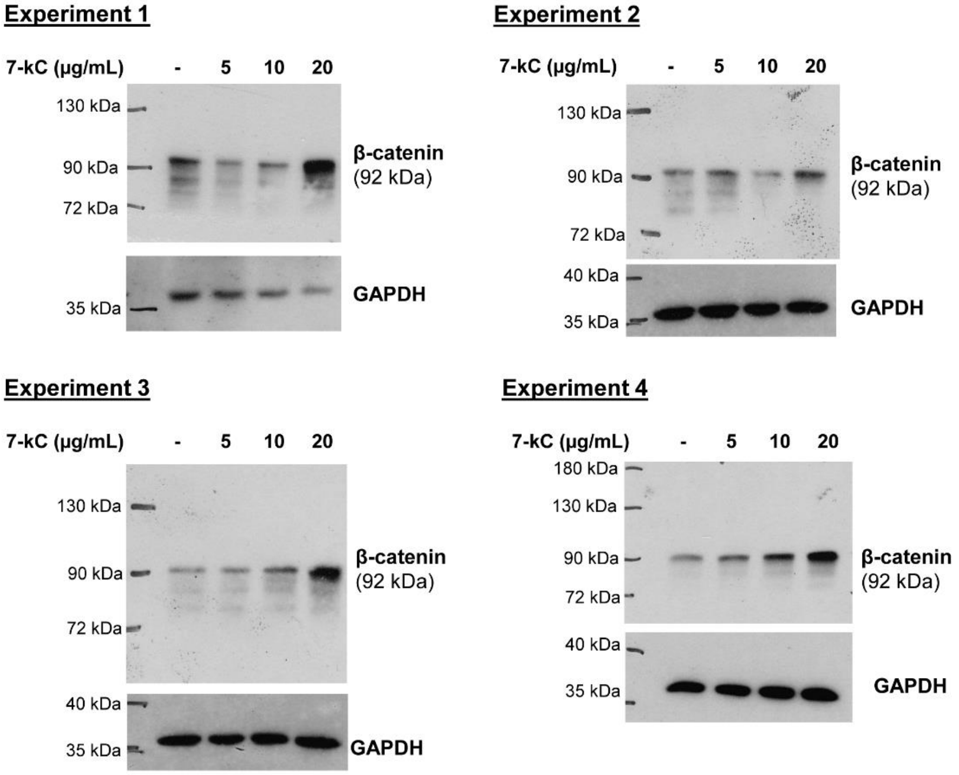

Table 6: Relative expression of **β-catenin** in 7-ketocholesterol (7-KC)-treated HAECs.

		Protein band intensity normalized to its respective GAPDH intensity in the same membrane **(a)**			
	**7-KC (µg/mL)**	-	5	10	20
Experiment 1	β-catenin **(a)**	331.20	151.60	198.20	499.50
	**a/b**	1.11	0.51	0.67	1.68
Experiment 2	β-catenin **(a)**	298.00	369.50	224.10	505.60
	**a/b**	1.00	1.24	0.75	1.70
Experiment 3	β-catenin **(a)**	228.70	261.20	388.30	641.50
	**a/b**	0.77	0.88	1.31	2.16
Experiment 4	β-catenin **(a)**	330.80	440.60	596.10	788.30
	**a/b**	1.11	1.48	2.01	2.65
	Average β-catenin of untreated control **(b)**	297.18			
Plot graph	Average of **(a/b)** from 4 experiments	1.00	1.03	1.18	2.05

The background-adjusted intensity values of β-catenin were first normalized to their respective GAPDH values of the same membrane. The normalized β-catenin was then expressed relative to the average normalized values obtained from untreated controls across four independent experiments and plotted accordingly.

(iii) VE-cadherin. Protein band intensity in **Table 7.**

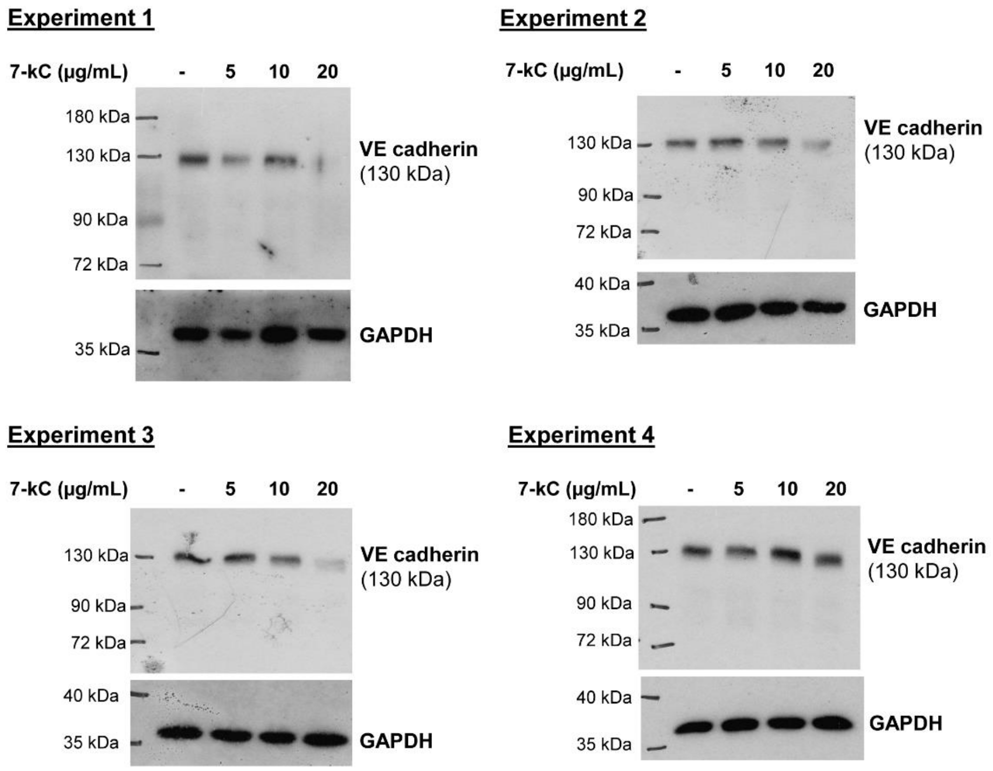

Table 7: Relative expression of **VE-cadherin** in 7-ketocholesterol (7-KC)-treated HAECs.

		Protein band intensity normalized to its respective GAPDH intensity in the same membrane **(a)**			
	**7-KC (µg/mL)**	-	5	10	20
Experiment 1	VE-cadherin **(a)**	699.90	386.80	681.00	218.30
	**a/b**	1.10	0.61	1.07	0.34
Experiment 2	VE-cadherin **(a)**	453.70	568.30	509.60	278.40
	**a/b**	0.72	0.90	0.80	0.44
Experiment 3	VE-cadherin **(a)**	694.80	722.30	588.00	162.60
	**a/b**	1.10	1.14	0.93	0.26
Experiment 4	VE-cadherin **(a)**	689.70	576.80	774.00	558.20
	**a/b**	1.09	0.91	1.22	0.88
	Average VE-cadherin of untreated control **(b)**	634.53			
Plot graph	Average of **(a/b)** from 4 experiments	1.00	0.89	1.01	0.48

The background-adjusted intensity values of VE-cadherin were first normalized to their respective GAPDH values of the same membrane. The normalized VE-cadherin was then expressed relative to the average normalized values obtained from untreated controls across four independent experiments and plotted accordingly.

EXCLI Journal 2024;23:1073-1090 – ISSN 1611-2156
Received: May 03, 2024, accepted: August 22, 2024, published: August 29, 2024

Supplement 4: Raw immunoblot images and protein band intensities of Figure 1D

Immunobloting analysis of the expression of (i) ICAM-1 and (ii) E-selectin was performed for 7-ketocholesterol (7-KC)-treated HAECs. Actin served as loading control. Densitometry analysis of the expression of protein was quantified using Quantity One software (Bio-Rad Laboratories, California, USA).

(i) ICAM-1. Protein band intensity in **Table 8**.

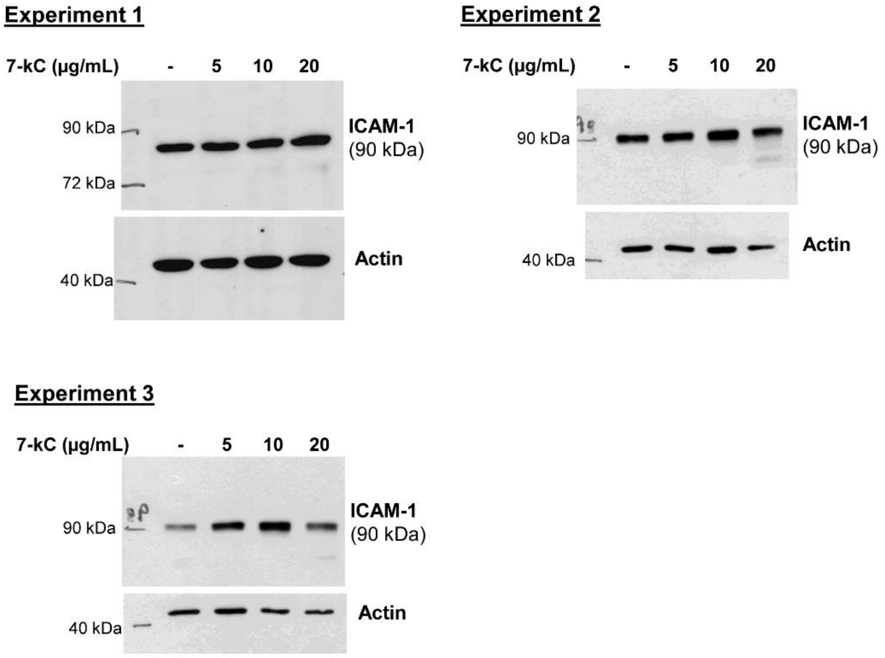

Table 8: Relative expression of **ICAM-1** in 7-ketocholesterol (7-KC)-treated HAECs.

		Protein band intensity normalized to its respective actin intensity in the same membrane **(a)**			
	**7-KC (µg/mL)**	-	5	10	20
Experiment 1	ICAM-1 **(a)**	1950.80	2173.70	2143.40	2811.90
	**a/b**	1.04	1.16	1.15	1.50
Experiment 2	ICAM-1 **(a)**	1673.30	2954.30	4814.40	3527.70
	**a/b**	0.90	1.58	2.57	1.89
Experiment 3	ICAM-1 **(a)**	1986.00	2256.50	2480.60	4022.50
	**a/b**	1.06	1.21	1.33	2.15
	Average ICAM-1 of un-treated control **(b)**	1870.03			
Plot graph	Average of **(a/b)** from 3 experiments	1.00	1.32	1.68	1.85

The background-adjusted intensity values of ICAM-1 were first normalized to their respective actin values of the same membrane. The normalized ICAM-1 was then expressed relative to the average normalized values obtained from untreated controls across three independent experiments and plotted accordingly.

EXCLI Journal 2024;23:1073-1090 – ISSN 1611-2156
Received: May 03, 2024, accepted: August 22, 2024, published: August 29, 2024

(ii) E-selectin. Protein band intensity in **Table 9.**

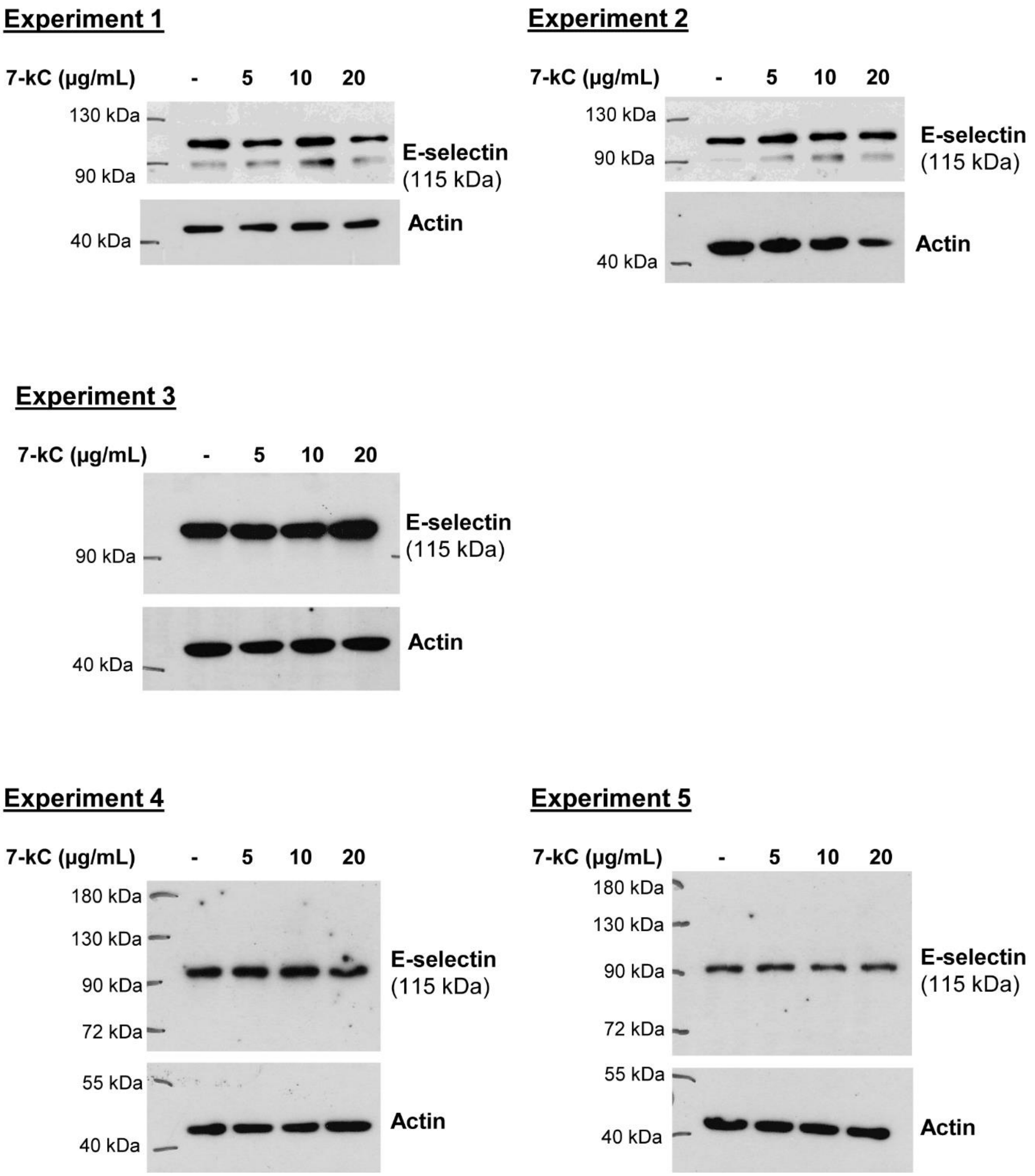

EXCLI Journal 2024;23:1073-1090 – ISSN 1611-2156
Received: May 03, 2024, accepted: August 22, 2024, published: August 29, 2024

Table 9: Relative expression of **E-selectin** in 7-ketocholesterol (7-KC)-treated HAECs.

		Protein band intensity normalized to its respective actin intensity in the same membrane **(a)**			
	**7-KC (µg/mL)**	-	5	10	20
Experiment 1	E-selectin **(a)**	1963.50	1588.30	1801.60	1833.10
	**a/b**	1.13	0.91	1.04	1.06
Experiment 2	E-selectin **(a)**	1675.00	1794.40	2219.50	2388.80
	**a/b**	0.96	1.03	1.28	1.37
Experiment 3	E-selectin **(a)**	1688.10	1885.40	2090.60	3297.90
	**a/b**	0.97	1.09	1.20	1.90
Experiment 4	E-selectin **(a)**	1745.40	2123.10	2105.20	2735.90
	**a/b**	1.00	1.22	1.21	1.57
Experiment 5	E-selectin **(a)**	1617.70	1175.70	1132.30	3236.90
	**a/b**	0.93	0.68	0.65	1.86
	Average E-selectin of untreated control **(b)**	1737.94			
Plot graph	Average of **(a/b)** from 5 experiments	1.00	0.99	1.08	1.55

The background-adjusted intensity values of E-selectin were first normalized to their respective actin values of the same membrane. The normalized E-selectin was then expressed relative to the average normalized values obtained from untreated controls across five independent experiments and plotted accordingly.

EXCLI Journal 2024;23:1073-1090 – ISSN 1611-2156
Received: May 03, 2024, accepted: August 22, 2024, published: August 29, 2024

Supplement 5: Raw immunoblot images and protein band intensities of Figure 2A
Immunobloting analysis of the expression of phosphorylated AKT [p-AKT (Ser473)] and total AKT was performed for 7-ketocholesterol (7-KC)-treated HAECs. GAPDH served as loading control. Densitometry analysis of the expression of protein was quantified using Quantity One software (Bio-Rad Laboratories, California, USA) and shown in **Table 10**.

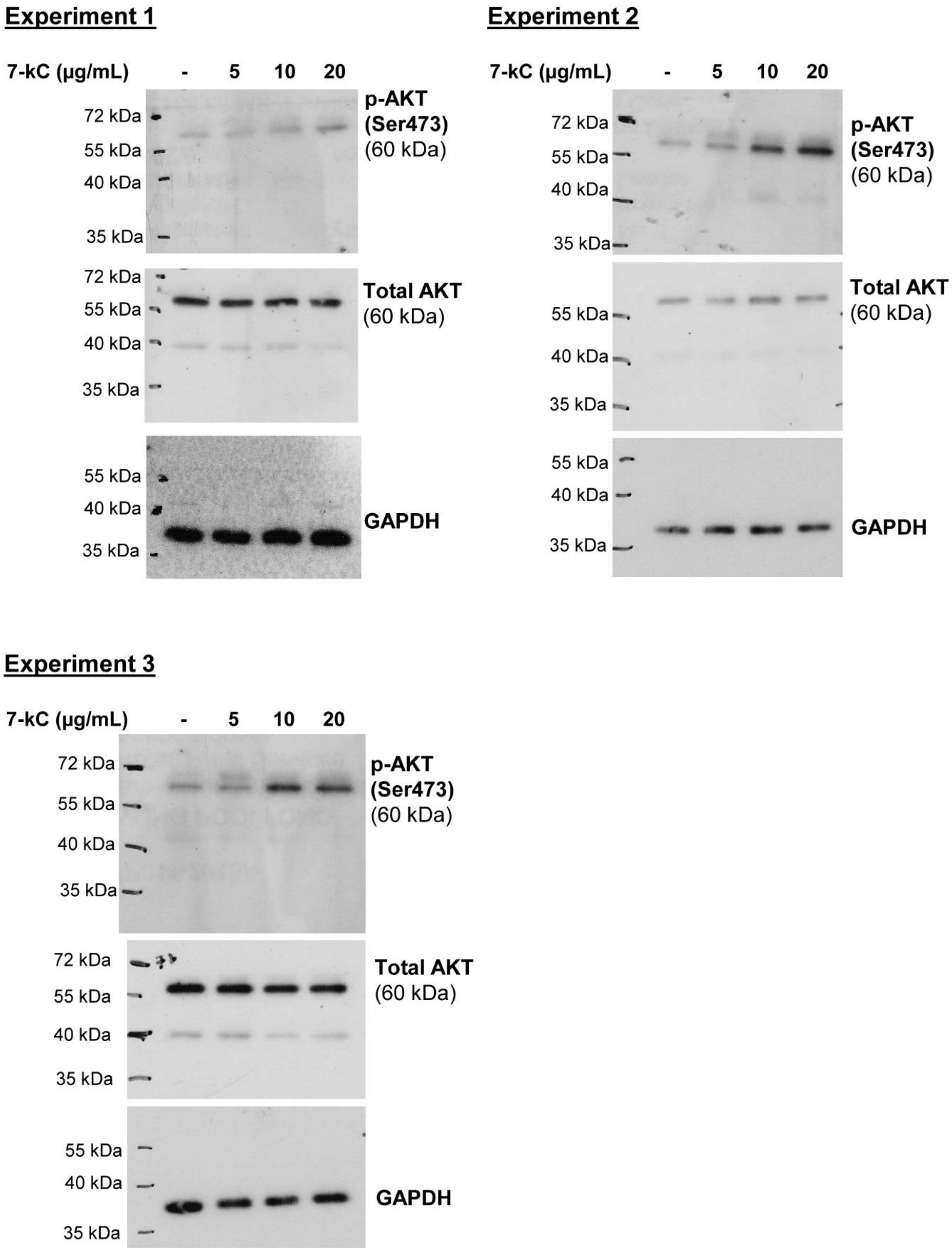

EXCLI Journal 2024;23:1073-1090 – ISSN 1611-2156
Received: May 03, 2024, accepted: August 22, 2024, published: August 29, 2024

Table 10: Expression of p-AKT (Ser473) relative to total AKT in 7-ketocholesterol (7-KC)-treated HAECs.

		Protein band intensity normalized to its respective GAPDH in the same membrane*			
	**7-KC (µg/mL)**	-	5	10	20
Experiment 1	p-AKT (Ser473)*	205.00	332.70	542.50	737.40
	AKT*	3632.20	3264.50	3271.60	3032.50
	p-AKT/AKT **(a)**	0.06	0.10	0.17	0.24
	**a/b**	0.15	0.27	0.44	0.65
Experiment 2	p-AKT (Ser473)*	30.60	65.50	137.30	220.20
	AKT*	36.50	31.70	60.50	49.80
	p-AKT/AKT **(a)**	0.84	2.07	2.27	4.42
	**a/b**	2.23	5.49	6.03	11.74
Experiment 3	p-AKT (Ser473)*	241.40	348.20	745.60	612.00
	AKT*	1027.80	964.90	809.30	862.70
	p-AKT/AKT **(a)**	0.23	0.36	0.92	0.71
	**a/b**	0.62	0.96	2.45	1.88
	Average p-AKT/AKT of untreated control **(b)**	0.38			
Plot graph	Average of **(a/b)** from 3 experiments	1.00	2.24	2.97	4.76

The background-adjusted intensity values of p-AKT (Ser 473) and total AKT were first normalized to their respective GAPDH values of the same membrane. Next, the ratio of normalized p-AKT (Ser473) to total AKT was calculated. This p-AKT (Ser473)/total AKT ratio was expressed relative to the average ratio obtained from untreated controls across three independent experiments and plotted accordingly.

EXCLI Journal 2024;23:1073-1090 – ISSN 1611-2156
Received: May 03, 2024, accepted: August 22, 2024, published: August 29, 2024

Supplement 6: Raw immunoblot images and protein band intensities of Figure 2B

Immunobloting analysis of the expression of phosphorylated AKT (p-AKT) (Ser473) and total AKT was performed for 7-ketocholesterol (7-KC)-treated HAECs with the presence of AKT inhibitor VII. GAPDH served as loading control. Densitometry analysis of the expression of protein was quantified using Quantity One software (Bio-Rad Laboratories, California, USA) and shown in **Table 11**.

Experiment 1

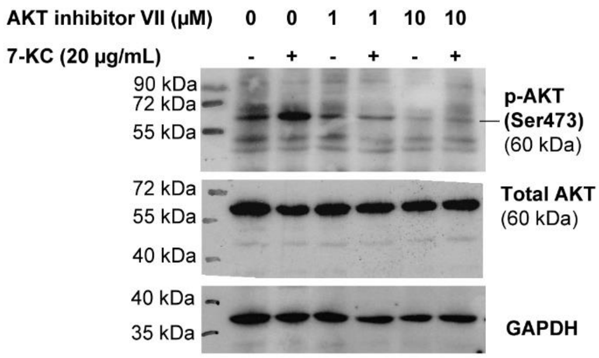

Experiment 2

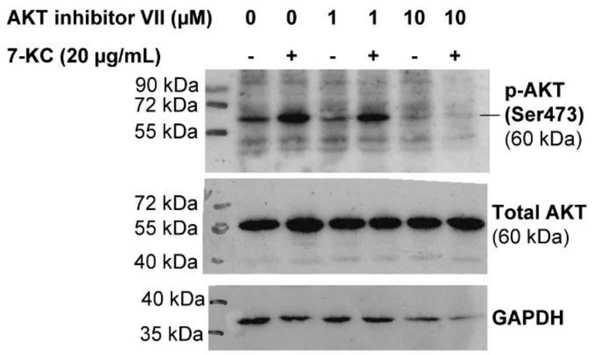

Experiment 3

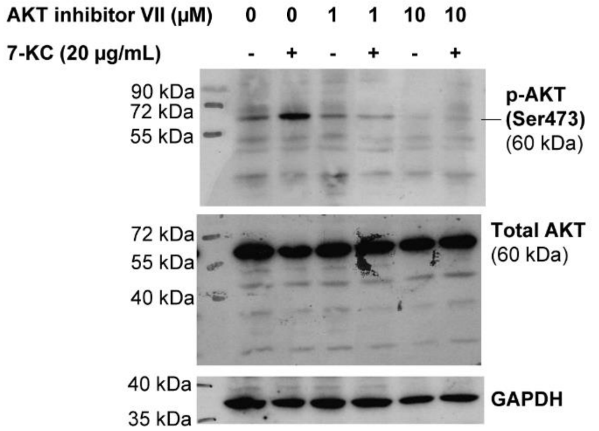

EXCLI Journal 2024;23:1073-1090 – ISSN 1611-2156
Received: May 03, 2024, accepted: August 22, 2024, published: August 29, 2024

Table 11: Expressions of p-AKT (Ser473) relative to total AKT in 7-ketocholesterol (7-KC)-treated HAECs with the presence of AKT inhibitor VII.

		Protein band intensity normalized to its respective GAPDH in the same membrane*					
	**AKT inhibitor VII (µM)**	0		1		10	
	**7-KC (20 µg/mL)**	-	+	-	+	-	+
Experiment 1	p-AKT (Ser473)*	882.70	1479.70	928.20	1334.60	1085.30	1281.90
	AKT*	1391.90	1666.60	1599.80	1562.00	2347.70	3360.20
	p-AKT/AKT **(a)**	0.63	0.89	0.58	0.85	0.46	0.38
	**a/b**	1.02	1.43	0.94	1.38	0.75	0.62
Experiment 2	p-AKT (Ser473)*	989.70	1511.50	934.90	1041.60	871.80	1088.50
	AKT*	1615.10	1567.30	1634.40	1776.80	2804.40	3432.50
	p-AKT/AKT **(a)**	0.61	0.96	0.57	0.59	0.31	0.32
	**a/b**	0.99	1.56	0.92	0.95	0.50	0.51
Experiment 3	p-AKT (Ser473)*	1118.60	1367.90	1001.80	1018.90	522.60	465.60
	AKT*	1823.40	1655.80	1769.70	1786.50	1823.60	1867.40
	p-AKT/AKT **(a)**	0.61	0.83	0.57	0.57	0.29	0.25
	**a/b**	0.99	1.33	0.91	0.92	0.46	0.40
	average p-AKT/AKT of untreated control **(b)**	0.62					
Plot graph	average of **(a/b)** from 3 experiments	1.00	1.44	0.92	1.08	0.57	0.51

The background-adjusted intensity values of p-AKT (Ser 473) and total AKT were first normalized to their respective GAPDH values of the same membrane. Next, the ratio of normalized p-AKT (Ser473) to total AKT was calculated. This p-AKT (Ser473)/total AKT ratio was then expressed relative to the average ratio obtained from untreated controls across three independent experiments and plotted accordingly.

EXCLI Journal 2024;23:1073-1090 – ISSN 1611-2156
Received: May 03, 2024, accepted: August 22, 2024, published: August 29, 2024

Supplement 7: Raw immunoblot images of Figure 4B with respective protein expression graphs of Figure 4C

Immunobloting analysis of the expression of (i) phosphorylated AKT [p-AKT (Ser473)], total AKT, (ii) VE-cadherin and (iii) E-selectin was performed for 7-ketocholesterol (7-KC)-treated miR-134-5p knockdown HAECs. Mock sample was HAECs treated with 0.1 % transfection reagent without miR-134-5p inhibitor. GAPDH served as loading control.

(i) Phosphorylated AKT (p-AKT). Protein intensity shown in **Table 12**.

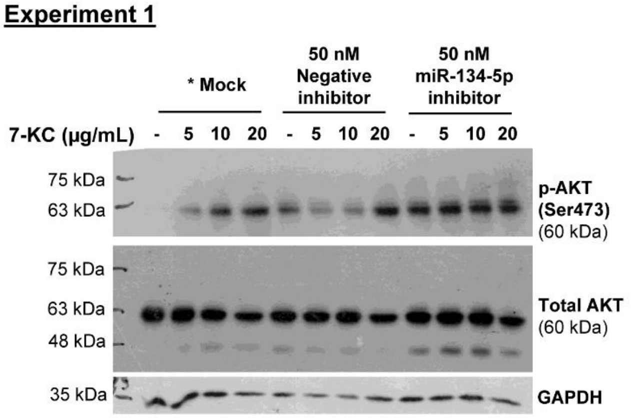

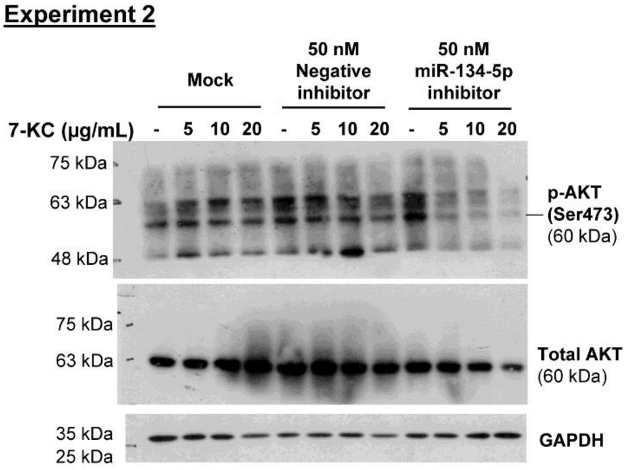

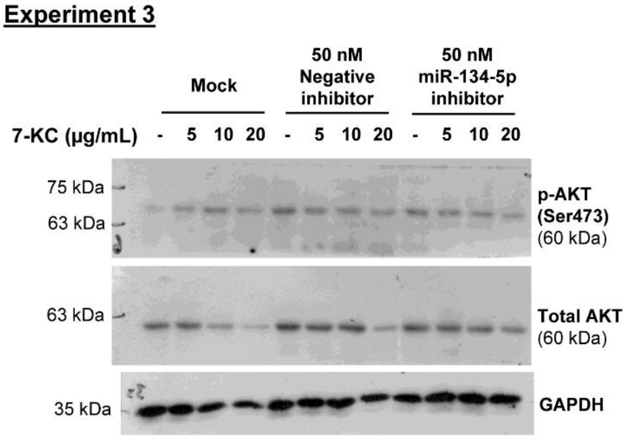

EXCLI Journal 2024;23:1073-1090 – ISSN 1611-2156
Received: May 03, 2024, accepted: August 22, 2024, published: August 29, 2024

Table 12: Expressions of p-AKT (Ser473) relative to total AKT in 7-ketocholesterol (7-KC)-treated miR-134-5p knockdown HAECs as shown in Figure 4C.

		Protein band intensity normalized to its respective GAPDH in the same membrane*							
		**50 nM negative inhibitor**				**50 nM miR-134-5p inhibitor**			
Experiment 1	**7-KC (µg/mL)**	-	5	10	20	-	5	10	20
	p-AKT (Ser473)*	517.60	306.80	238.50	1395.20	1684.80	1400.70	2009.90	2325.70
	AKT*	1693.80	2264.20	2305.50	1162.70	1197.50	1363.30	1214.50	1419.50
	p-AKT/AKT	0.31	0.14	0.10	1.20	1.41	1.03	1.65	1.64
Experiment 2	p-AKT (Ser473)*	1268.70	1124.50	1119.80	1299.70	2590.00	1390.90	926.10	532.60
	AKT*	2120.00	2231.30	2198.70	1802.10	2372.80	2226.80	1477.70	1268.00
	p-AKT/AKT	0.60	0.50	0.51	0.72	1.09	0.62	0.63	0.42
Experiment 3	p-AKT (Ser473)*	417.20	315.80	324.70	328.50	334.50	236.70	215.30	162.60
	AKT*	608.70	512.70	532.10	234.60	459.10	541.90	410.00	329.80
	p-AKT/AKT	0.69	0.62	0.61	1.40	0.73	0.44	0.53	0.49
Plot graph	Average p-AKT/AKT	0.53	0.42	0.41	1.11	1.08	0.70	0.94	0.85

The background-adjusted intensity values of p-AKT (Ser473) and total AKT were first normalized to their respective GAPDH values of the same membrane. Next, ratio of normalized p-AKT (Ser473) to total AKT from three independent experiments were calculated and plotted. Mock-transfected data were not plotted.

EXCLI Journal 2024;23:1073-1090 – ISSN 1611-2156
Received: May 03, 2024, accepted: August 22, 2024, published: August 29, 2024

(ii) VE-cadherin. Protein band intensity shown in **Table 13**.

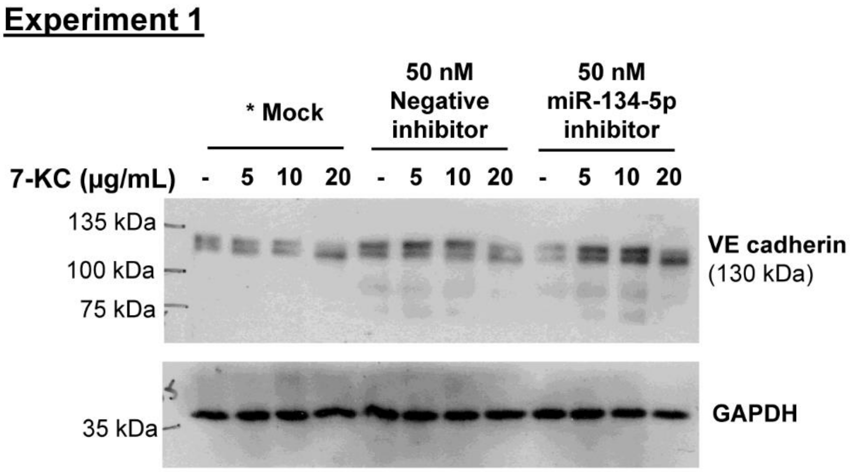

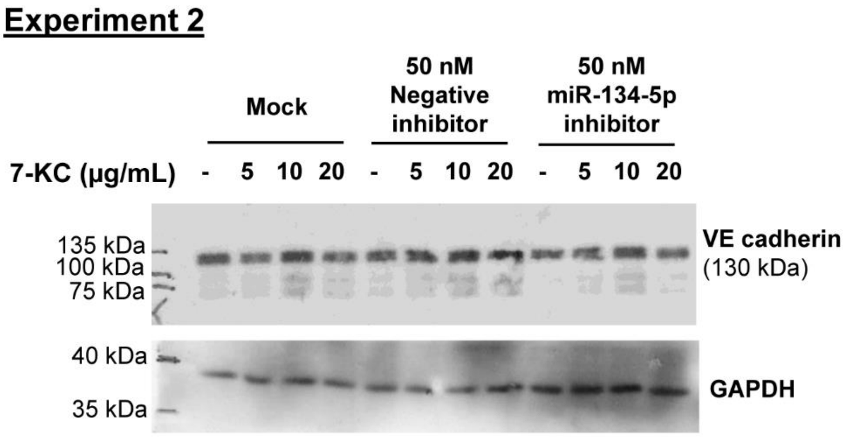

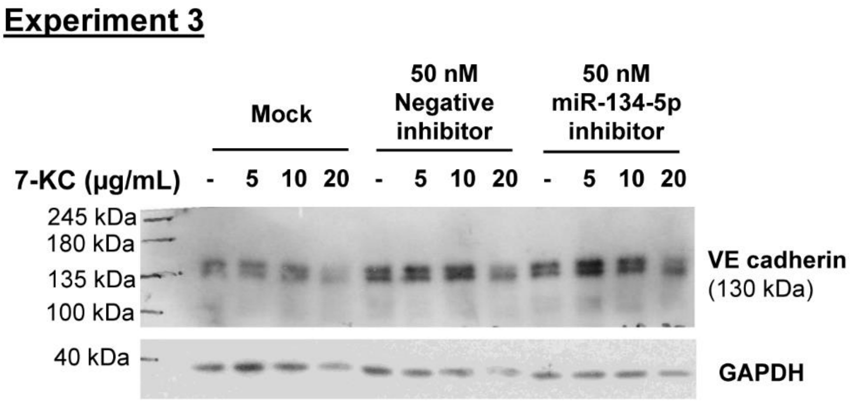

EXCLI Journal 2024;23:1073-1090 – ISSN 1611-2156
Received: May 03, 2024, accepted: August 22, 2024, published: August 29, 2024

Table 13: Normalized expressions of VE-cadherin in 7-ketocholesterol (7-KC)-treated miR-134-5p knockdown HAECs as shown in Figure 4C.

		Protein band intensity normalized to its respective GAPDH intensity in the same membrane*							
		**50 nM negative inhibitor**				**50 nM miR-134-5p inhibitor**			
	**7-KC (µg/mL)**	-	5	10	20	-	5	10	20
Experiment 1	VE-Cadherin*	927.30	1440.40	1309.60	498.70	283.40	814.40	818.40	964.50
Experiment 2	VE-Cadherin*	977.10	1446.70	1262.40	697.70	505.10	417.30	563.80	543.40
Experiment 3	VE-Cadherin*	654.40	896.20	1078.40	513.30	780.20	1165.80	981.60	1094.40
Plot graph	Average VE-Cadherin	852.93	1261.10	1216.80	569.90	522.90	799.17	787.93	867.43

The background-adjusted intensity values of VE-cadherin were first normalized to their respective GAPDH values of the same membrane. The average normalized VE-cadherin expressions from three independent experiments were calculated and plotted. Mock-transfected data were not plotted.

EXCLI Journal 2024;23:1073-1090 – ISSN 1611-2156
Received: May 03, 2024, accepted: August 22, 2024, published: August 29, 2024

(iii) E-selectin. Protein band intensity shown in **Table 14**.

Experiment 1

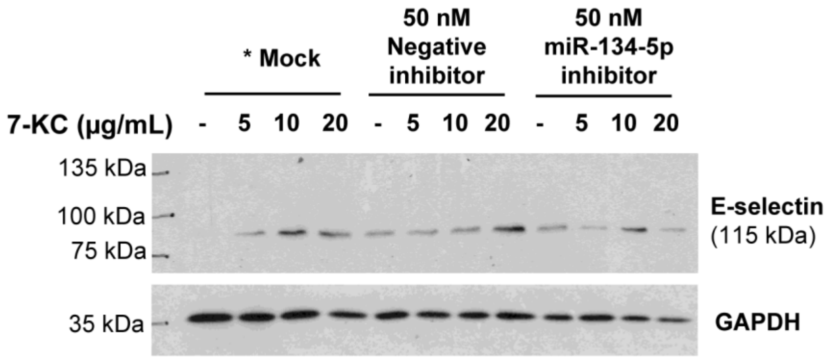

Experiment 2

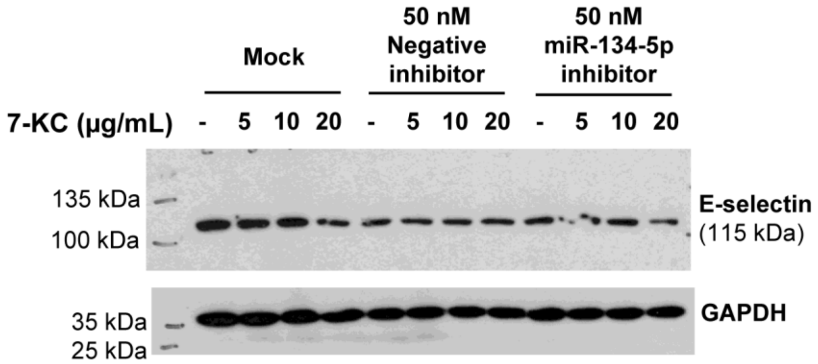

Experiment 3

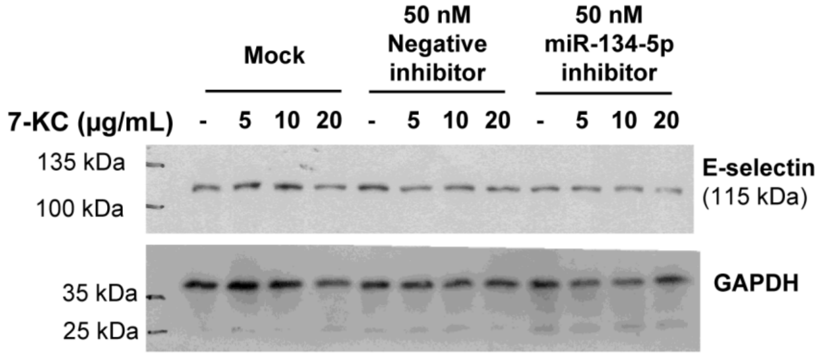

EXCLI Journal 2024;23:1073-1090 – ISSN 1611-2156
Received: May 03, 2024, accepted: August 22, 2024, published: August 29, 2024

Table 14: Normalized expressions of E-selectin in 7-ketocholesterol (7-KC)-treated miR-134-5p knockdown HAECs as shown in Figure 4C.

	Protein band intensity normalized to its respective GAPDH intensity in the same membrane							
	**50 nM negative inhibitor**				**50 nM miR-134-5p inhibitor**			
**7-KC (µg/mL)**	-	5	10	20	-	5	10	20
Experiment 1	427.30	422.30	547.00	1058.70	672.40	193.40	934.70	409.10
Experiment 2	619.40	568.60	633.70	679.00	644.80	471.70	758.30	492.00
Experiment 3	654.00	508.20	616.00	1060.00	449.20	324.10	206.50	222.60
Average (plot graph)	566.90	499.70	598.90	932.57	588.80	329.73	633.17	374.57

The background-adjusted intensity values of E-selectin were first normalized to their respective GAPDH values of the same membrane. The average normalized E-selectin expressions from three independent experiments were calculated and plotted. Mock-transfected data were not plotted.

EXCLI Journal 2024;23:1073-1090 – ISSN 1611-2156
Received: May 03, 2024, accepted: August 22, 2024, published: August 29, 2024

Supplement 8: Raw immunoblot images and protein band intensities of Figure 4D

Immunobloting analysis of the expression of phosphorylated eNOS [p-eNOS (S1177)] and eNOS was performed for 7-ketocholesterol (7-KC)-treated miR-134-5p knockdown HAECs. Negative control inhibitor served as non-targeting control. GAPDH served as loading control. Densitometry analysis of the expression of protein was quantified using Quantity One software (Bio-Rad Laboratories, California, USA) and shown in **Table 15.**

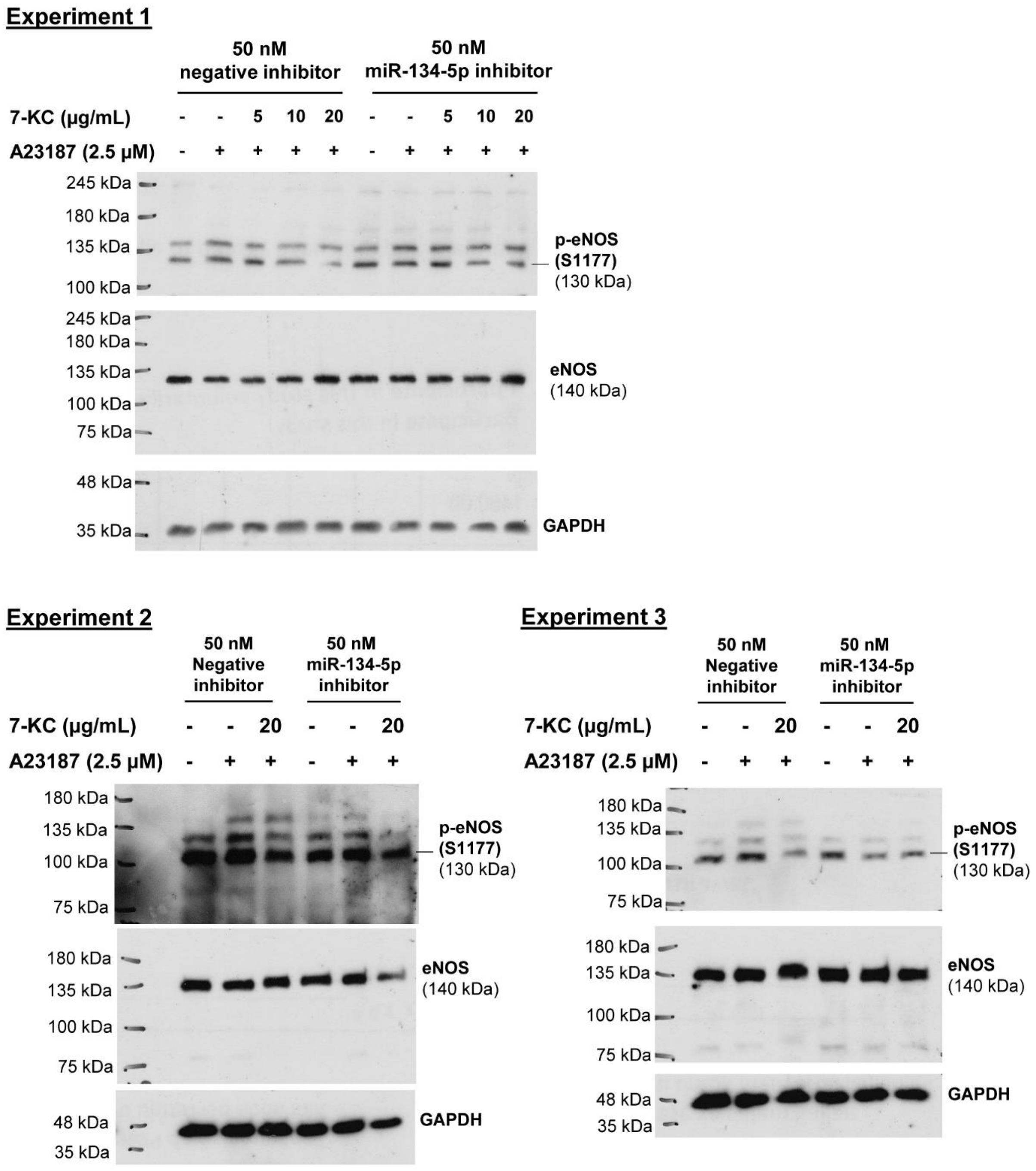

EXCLI Journal 2024;23:1073-1090 – ISSN 1611-2156
Received: May 03, 2024, accepted: August 22, 2024, published: August 29, 2024

Table 15: Expressions of p-eNOS (S1177) relative to total eNOS in 7-ketocholesterol (7-KC)-treated miR-134-5p knockdown HAECs.

		Protein band intensity normalized to its respective GAPDH in the same membrane*					
		**50 nM negative inhibitor**			**50 nM miR-134-5p inhibitor**		
	**7-KC (µg/mL)**	-	-	20	-	-	20
	**A23187 (2.5 µM)**	-	+	+	-	+	+
Experiment 1	p-eNOS (S1177)*	0.75	1.19	0.35	1.14	1.33	0.77
	eNOS*	0.62	0.75	1.31	1.31	1.44	1.12
	p-eNOS / eNOS	1.21	1.59	0.27	0.87	0.92	0.69
Experiment 2	p-eNOS (S1177)*	0.82	0.73	0.57	0.57	0.73	1.04
	eNOS*	0.79	1.00	0.83	0.78	0.91	0.80
	p-eNOS / eNOS	1.04	0.73	0.68	0.73	0.81	1.30
Experiment 3	p-eNOS (S1177)*	0.82	1.19	0.57	0.88	0.51	0.63
	eNOS*	0.84	0.89	1.01	0.85	0.88	0.94
	p-eNOS / eNOS	0.97	1.33	0.56	1.04	0.58	0.67
Plot graph	average p-eNOS/eNOS	1.07	1.22	0.50	0.88	0.77	0.89

The background-adjusted intensity values of p-eNOS (S1177) and total eNOS were first normalized to their respective actin values of the same membrane. Next, the ratio of normalized p-eNOS (S1177) to total eNOS was calculated. This p-eNOS (S1177)/total eNOS ratio was then expressed relative to the average ratio obtained from untreated controls across four independent experiments and plotted accordingly.

EXCLI Journal 2024;23:1073-1090 – ISSN 1611-2156
Received: May 03, 2024, accepted: August 22, 2024, published: August 29, 2024

Supplement 9: Raw immunoblot images and protein band intensities of Figure 5D

Immunobloting analysis of the expression of FOXM1 was performed for HUVECs transfected with 50 nM biotinylated-miR-134-5p (bi-miR-134-5p) mimic or bi-cel-miR-67 mimic. HUVECs transfected with bi-cel-miR-67 mimic served as non-targeting control. GAPDH served as loading control. Densitometry analysis of the expression of protein was quantified using Quantity One software (Bio-Rad Laboratories, California, USA) and shown in **Table 16**.

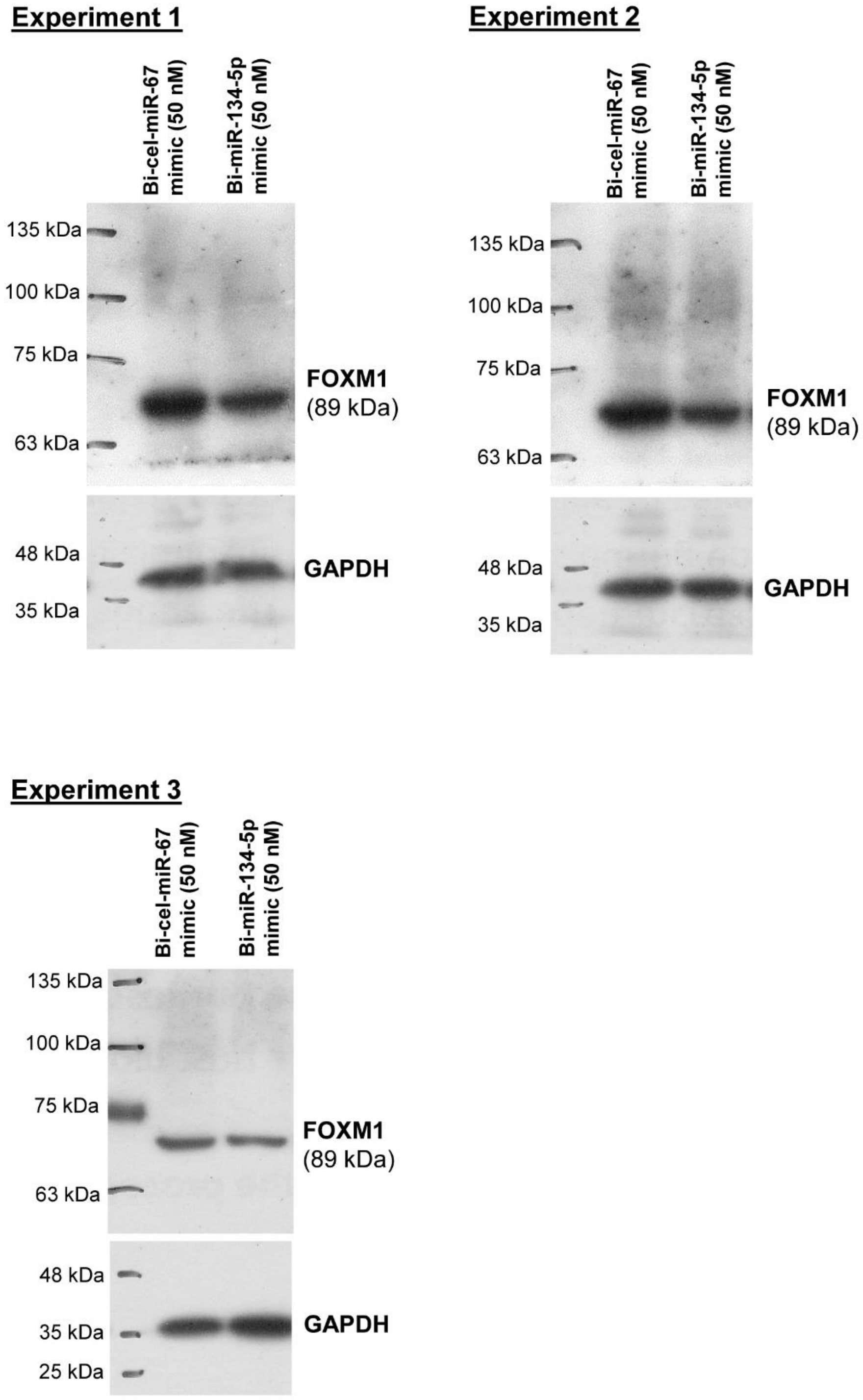

EXCLI Journal 2024;23:1073-1090 – ISSN 1611-2156
Received: May 03, 2024, accepted: August 22, 2024, published: August 29, 2024

Table 16: Relative expression of **FOXM1** in HUVECs transfected with biotinylated-miR-134-5p (bi-miR-134-5p) mimic or bi-cel-miR-67 mimic.

		**Bi-cel-miR-67 mimic (50 nM)**	**Bi-miR-134-5p mimic (50 nM)**
Experiment 1	FOXM1 **(a)**	0.708	0.524
	**a/b**	1.02	0.75
Experiment 2	FOXM1 **(a)**	0.679	0.470
	**a/b**	0.98	0.68
Experiment 3	FOXM1 **(a)**	0.698	0.424
	**a/b**	1.00	0.61
	Average FOXM1 of untreated control **(b)**	0.695	
Plot graph	Average of **(a/b)** from 3 experiments	1	0.68

The background-adjusted intensity values of FOXM1 were first normalized to their respective GAPDH values of the same membrane. The normalized expressions of FOXM1 of HUVECs transfected with bi-miR-134-5p mimic were then compared those of non-targeting control (bi-cel-miR-67 mimic).
